# Predicting Toxicity toward Nitrifiers by Attention-Enhanced Graph Neural Networks and Transfer Learning from Baseline Toxicity

**DOI:** 10.1021/acs.est.4c12247

**Published:** 2025-02-27

**Authors:** Kunyang Zhang, Philippe Schwaller, Kathrin Fenner

**Affiliations:** † Department of Environmental Chemistry, Eawag, 8600 Dübendorf, Switzerland; ‡ Department of Chemistry, University of Zürich, 8057 Zürich, Switzerland; § Laboratory of Artificial Chemical Intelligence, Institute of Chemical Sciences and Engineering, EPFL, 1015 Lausanne, Switzerland; ∥ National Centre of Competence in Research Catalysis, EPFL, 1015 Lausanne, Switzerland

**Keywords:** transfer learning, interpretability, toxicity screening, structural alerts, nitrifiers

## Abstract

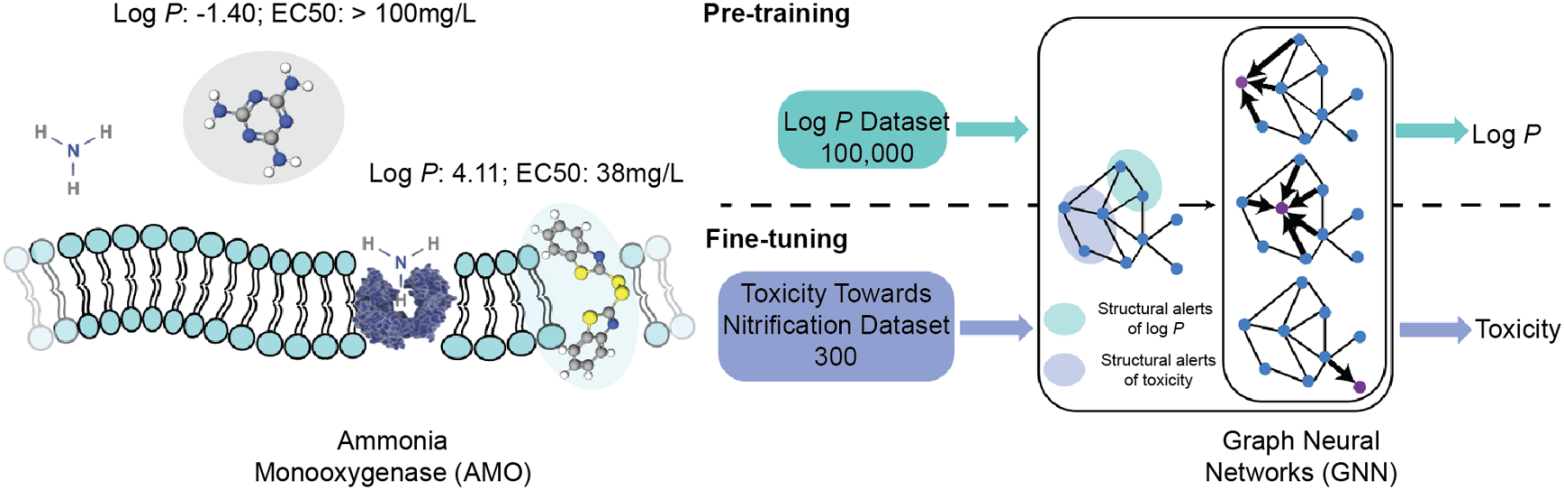

Assessing chemical environmental impacts is critical but challenging due to the time-consuming nature of experimental testing. Graph neural networks (GNNs) support superior prediction performance and mechanistic interpretation of (eco-)toxicity data, but face the risk of overfitting on the typically small experimental data sets. In contrast to purely data-driven approaches, we propose a mechanism-guided transfer learning strategy that is highly efficient and provides key insights into the underlying drivers of (eco-)toxicity. By leveraging the mechanistic link between baseline toxicity and toxicity toward nitrifiers, we pretrained a GNN on lipophilicity data (log P) and subsequently fine-tuned it on the limited data set of toxicity toward nitrifiers, achieving prediction performance comparable with pretraining on much larger but mechanistically less relevant data sets. Additionally, we enhanced GNN interpretability by adjusting multihead attentions after convolutional layers to identify key substructures, and quantified their contributions using a Shapley Value method adapted for graph-structured data with improved computational efficiency. The highlighted substructures aligned well with and effectively distinguished known structural alerts for baseline toxicity and specific modes of toxic action in nitrifiers. The proposed strategy will allow uncovering new structural alerts in other (eco)toxicity data, and thus foster new mechanistic insights to support chemical risk assessment and safe-by-design principles.

## Abstract 2

Predictive models for assessing environmental impacts are prone to overfitting when trained on small datasets. This study reports the application of transfer learning from baseline toxicity prediction to specific modes of action.

## Introduction

The evaluation of the environmental impacts of chemicals, including pesticides and their transformation products, presents a critical challenge for environmental studies, largely due to the labor-intensive and time-consuming nature of experimental toxicity assessment.^1,2^ For example, evaluating the toxicity of chemicals on microbial nitrification by ammonia-oxidizing bacteria (AOB) through *in vitro* methods typically takes more than a month.^3^ This complexity has led to a highly problematic discrepancy between the vast amount of commercially available chemicals with potential environmental impacts and the relatively limited number of chemicals that have been thoroughly evaluated.^4−6^ To address this issue, *in silico* models developed through machine learning are being promoted as a faster, less expensive, and mechanistically more relevant approach than experimental testing.^2,7^ Specifically, deep learning (DL) approaches such as Graph Neural Networks (GNNs) have emerged as a promising solution for developing *in silico*models since they offer both superior performance over classical machine learning models and improved mechanistic interpretability through learnable representations.^8,9^ The latter is of key importance to ensure that the reasoning behind predictions is consistent with established mechanistic knowledge or to gain novel insights into structural factors influencing environmental fate and effects of chemicals. However, the applicability of such advanced DL techniques in environmental chemistry and (eco-)toxicology is often hindered by the small sizes of experimental data sets,^10^ causing risks of overfitting and suboptimal predictive performance.^11^ The limited data availability prevents the model from learning complex patterns and accurately predicting outcomes, emphasizing the need for strategies like transfer learning to harness the full potential of GNNs in environmental chemistry and (eco-)toxicology. A recent related study successfully applied purely data-driven transfer learning with PubChem compounds to pretrain image-based DL models, followed by fine-tuning on small contaminant property data sets, achieving outstanding predictive performance.^12^

Microbial nitrification, particularly within soil ecosystems, plays an important role in nitrogen cycling, essential for plant growth and ecosystem health.^13^ Yet, the scarcity of experimental data is a challenge for developing DL models (i.e., GNNs) that capture the complex interactions between chemical structures and toxicity toward nitrifying microorganisms, known as nitrifiers. In contrast, there are plenty of experimental records for the octanol–water partition coefficient, i.e., log *P*, of chemicals, a physicochemical property often used as a surrogate measure to describe the ability of chemicals to partition into biological membranes. Log *P* values are therefore widely used as predictors of baseline toxicity,^14−16^ which describes a general mode of toxic action where a chemical nonspecifically disrupts cell membrane integrity, and, in doing so, interferes with a number of membrane-associated processes such as energy transduction, transport in and out of the cell, or enzyme activities.^17^ Microbial nitrification, in turn, is controlled by ammonia monooxygenase (AMO),^18^ which is a membrane-bound enzyme with its cellular location confirmed through electron microscopic immunocytochemistry.^19^ It is therefore reasonable to suspect that microbial nitrification may also suffer from membrane disturbance by baseline toxicants, in addition to more specific effects resulting from direct interactions of chemical substances with AMO, such as competitive inhibition of the enzyme’s active site^20^ or chelation of copper present in the enzyme’s active site.^21^

Attention-based methods show great promise in enhancing model interpretability by highlighting relevant features or substructures of the molecule that are pivotal in decision-making processes.^22^ However, the reliability of these methods as interpretability tools is not always guaranteed, as attention scores can sometimes mislead by attributing importance to features in a manner that is inconsistent with the underlying chemistry.^23^ For example, in GNNs, this could be caused by the information exchange among atoms during the message-passing phase, which alters the composition of each atom, as shown in Figure S8 of Supporting Information (SI). Consequently, global attention scores calculated for the updated atom features may not accurately represent the importance of the atoms in their original states. Correcting global attention scores for the original states of atoms by tracking the dynamic changes of atom features during the message-passing phase could be a solution. In addition, even if important substructures can be successfully identified, it is challenging to understand how they contribute to prediction since high attention scores do not straightforwardly translate into understanding how chemical structures impact the final prediction due to the complexity introduced by subsequent fully connected layers.^24^ Such complexity obscures the direct link between attention mechanisms and predictive outcomes, making it difficult to interpret the reasoning of GNN-based decision-making. A promising solution to this problem lies in the application of Shapley values,^25^ a concept from cooperative game theory. Already widely used for models handling data represented by a series of numeric features, the Shapley value method has recently been tailored for graph-structured data, which better captures the interconnected nature of graphs by analyzing subgraph structures as cohesive units.^26^ However, since enumerating substructures in a graph is an *O*(2^*n*^) problem and can be computationally intense, calculating Shapley values for all possible substructures in a molecule might suffer from a long computation time. Therefore, using corrected attention scores to identify key substructures and calculating Shapley values exclusively for these will substantially improve calculation efficiency.

In this study, we aimed to develop an efficient modeling strategy that provides optimal performance in predicting (eco-)toxicological endpoints subject to small data set limitations while affording interpretability in line with and beyond our current mechanistic understanding of toxicity toward nitrifiers. We addressed this aim by profiting from our existing understanding of baseline toxicity as an important driver of (eco-)toxicological effects. More concretely, we pretrained a GNN on a log *P* data set containing nearly 100,000 compounds, enabling the model to learn baseline toxicity, and then fine-tuned the model on the much smaller data set on toxicity toward nitrifiers as an illustrative example of a highly relevant, yet under-researched endpoint in chemical risk assessment.^27,28^ To ensure interpretability, our study further introduces an innovative approach to adjust multihead attention for the dynamic evolution of feature representations of atoms during message passing in GNNs. This adjustment ensures that attention scores accurately reflect the significance of atoms in their original states. We combined the adjusted attention method with Shapley values for graph data to efficiently interpret the model and finally analyzed the highlighted structural alerts in terms of their contribution to baseline toxicity and specific toxicity toward nitrifiers.

## Materials and Methods

### Graph Attention Networks

**Figure 1:**
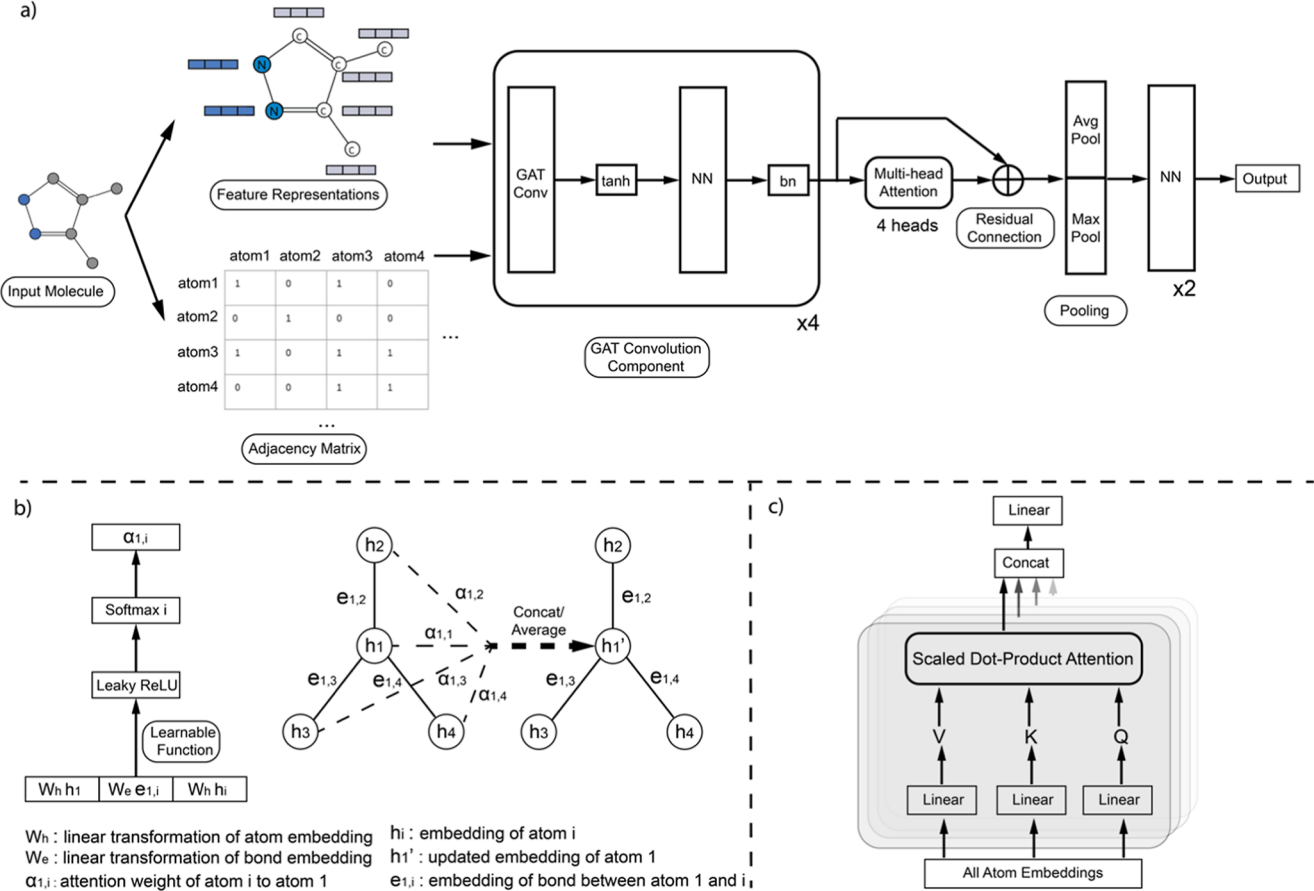
Overview of the architecture and attention mechanisms of the model. (a) Input molecule is converted into feature representations and an adjacency matrix for convolution operations, followed by a multihead attention component with four attention heads designed to capture the global attention of the input molecule. (b) Example of embedding updates for atom 1. The local attention weight is calculated for each neighbor, and the updated embedding is a weighted aggregation of the neighboring embeddings, including that of atom 1 itself. (c) Multihead attention component, wherein the atom embeddings are converted to Value (*V*), Key (*K*), and Query (*Q*) through linear transformation, and the attentions are calculated as scaled dot products. The attentions from each head are concatenated and subsequently transformed by a linear transformation to align with the dimension of the atom embeddings.

We introduced a GNNs framework tailored for predicting molecular properties through graph representations with a particular emphasis on the strategic application of attention mechanisms, as shown in Figure 1. A key advantage of GNNs lies in their ability to evolve the feature representation of each node by considering the attributes and relational dynamics of its neighbors, which is crucial for capturing complex relationships within the graph structure.^29^ The evolution process of feature representation is called message passing. In our model, message passing was conducted through sequential Graph Attention Networks Convolution (GATConv) layers of PyTorch.^30^ For each atom, convolution layers computed attention coefficients for each of its neighboring atoms via a learnable function, aiming to focus more on the most relevant neighbors. This localized attention mechanism enables an effective aggregation of neighborhood features sensitive to the molecule’s topology. To complement the localized attention, we developed a multihead attention layer^31^ that, following the GATConv layers, shifted the focus from neighboring atoms to the entire molecule. This architecture allowed the model to capture global relationships and interactions within the molecule. The final embedding of a molecule was achieved by aggregating features from each node through concatenated global maximum pooling and global average pooling, followed by two fully connected layers for the regression or classification of molecular properties.

The transformation of Simplified Molecular Input Line Entry System (SMILES) strings into graph representations was accomplished using the featurizer^32^ from DeepChem^33^ designed for the general graph convolution networks of molecules. Node (i.e., atom) and edge (i.e., bond) features were extracted by the featurizer and served as the model input, along with the adjacency matrix of atoms. Specifically, node features contained atom type, formal charge, hybridization type, hydrogen bonding, aromaticity, degree, number of hydrogens, chirality, and partial charge. For bond features, the values corresponded to bond type, ring, conjugation, and stereo configuration.

### Collection of Toxicity Data on Nitrifiers

To fine-tune the model for the prediction of toxicity toward nitrifiers, we curated a comprehensive data set derived from an extensive literature review, which consists of industrially significant compounds, patented nitrification inhibitors, and chemicals with structures similar to known nitrification inhibitors. This data set also includes biological nitrification inhibitors (i.e., chemical compounds released by plants and microorganisms) and known substrates of AMO capable of competitively inhibiting ammonia oxidation. Additionally, it contains agrochemicals, including herbicides, fungicides, and insecticides. Regarding the compound space of the data set, it covers a wide range of chemical structures, including aliphatic and aromatic amines, sulfur-containing compounds, acetylenic compounds, heterocyclic nitrogen compounds, and 1,3,5-triazines with various substituents. Data for a total of 288 compounds were collected. The compounds that inhibited nitrification by more than 50% at concentrations of less than 80 mg/L were labeled as positive in the data set, while the remaining compounds were labeled as negative. This threshold was chosen to ensure a relatively balanced ratio of positive to negative samples (i.e., 172:116). Details of the data set are provided in SI Section S2. The data set was initially split into 10% for testing and 90% for training and validation. The combined training and validation data were used for a 10-fold cross validation to optimize hyperparameters. After hyperparameter tuning, we conducted a total of 10 independent train-test splits (including the initial split), where the data set was repeatedly split into 90% training and 10% test, and performance was averaged over these splits to ensure a robust evaluation.

### Attention Correction

During the message-passing phase within the GATConv layers, the feature representation of each atom was iteratively updated through the aggregation of feature information from its immediate neighbors. This process was applied across multiple GATConv layers, resulting in progressive modification of the feature representations to reflect the influence of the topological environment of the molecule. The calculation of the matrix of global attention weights (i.e., *A*_*i*_) is depicted in SI Section S3. To precisely attribute attention weights to the corresponding original atoms, we explored the aggregation process inside each GATConv layer to decompose the final aggregated feature representations. The attention weight matrix in the *l*-th GATConv layer was expressed as *C*_*l*_, reflecting the contribution of each atom to the weighted feature sum. The overall composition of atom representations through the model (i.e., *C*) was computed as the successive product of attention weight matrices across all included GATConv layers (eq 1).
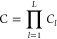
1In alignment with the residual connectivity scheme employed in the global multihead attention component, the averaged multihead attention, computed as the mean of attention weights (i.e., *A*_*i*_) across *N* (*N* = 4) different attention heads, was first multiplied with the composite atom representation *C* and then added to *C*, thereby maintaining congruence with the residual connectivity scheme (eq 2).
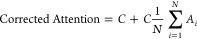
2

### Shapley Values for Key Substructure Identification

In this work, the Shapley value method adapted from previous studies^26,34^ was utilized to quantify the contribution of important substructures, identified by corrected global attentions, toward predictive outcomes. For a given molecule *S* containing *m* atoms, alongside our trained predictive model , we focused on the computation of the Shapley value for a targeted substructure *S*_*i*_ with *k* atoms. Let *V* = {υ_1_,υ_2_,···,υ_*m*_} represent all atoms within molecule *S*, with the set {υ_1_,···,υ_*k*_} representing the atoms in *S*_*i*_, and the remaining atoms {υ_*k*+1_,···,υ_*m*_} constituting the complement set of *S*_*i*_ in *S*. Similarly to the previous study,^26^ to obtain both good approximation and computational efficiency, the Shapley value of a substructure was calculated only with the atoms and bonds that can be reached by the substructure within *L* bonds (*L* = 4). If there were γ (γ ≤ *m*–*k*) atoms reachable from *S*_*i*_ within L bonds, the set of players was denoted as *T* = {*S*_*i*_,υ_*k*+1_,···,υ_*r*_}, where the substructure *S*_*i*_ was treated as a single player. Additionally, *E* represents the ensemble of all possible coalitions among the players. The Shapley value for substructure *S*_*i*_ was then calculated as follows
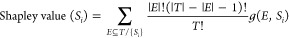
3
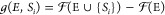
4

Atoms and bonds not included in the coalition *E* and substructure *S*_*i*_ were masked with zero features rather than removed from the molecular structure. This technique preserved the original structure of molecule while effectively mitigating the influence of irrelevant parts. To further improve the computational efficiency of Shapley value estimation, our method employed a parallel processing strategy, which simultaneously calculated the marginal contributions  of multiple coalitions *E*. In addition, Shapley values were calculated for only the important substructures identified by the adjusted attention mechanisms to improve computation efficiency.

## Results and Discussion

### Graph Attention Networks for Lipophilicity Prediction

Before training the model with log *P* and nitrification toxicity data sets, its performance was tested with other publicly available data sets, including solubility (i.e., ESOL),^33^ mutagenicity,^35^ hERG,^36^ and BBBP.^37^ The performance is comparable to that of GNNs with more complex architectures,^9^ and detailed results on those data sets are provided in SI Section S1.3.

**Figure 2:**
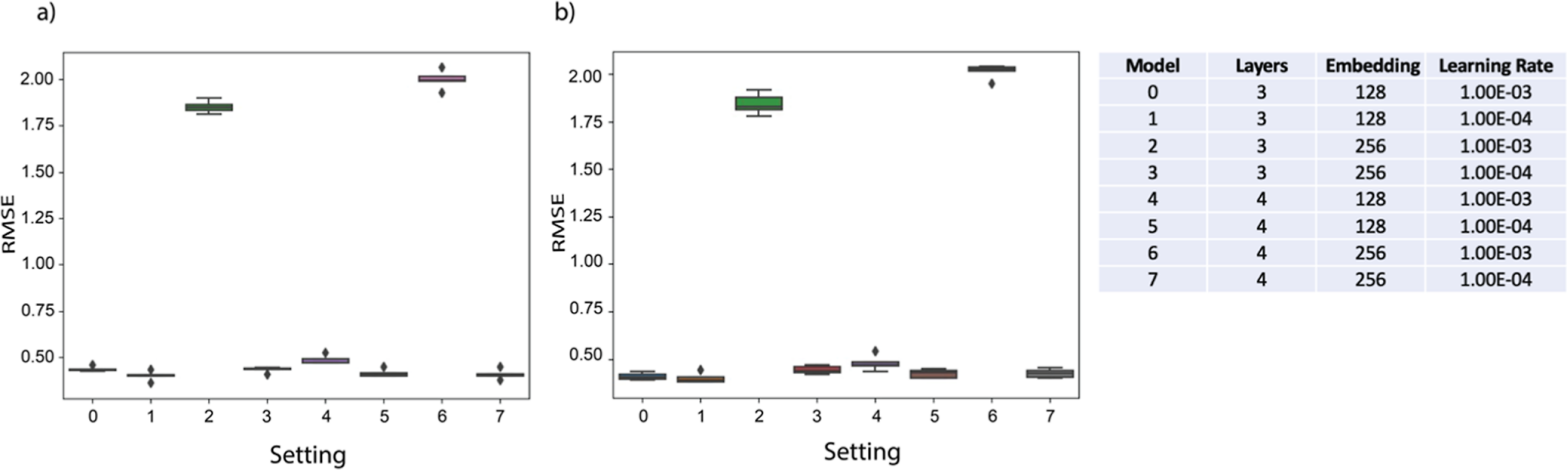
Log *P* prediction performance of (a) original and (b) tautomers SMILES with four attention heads and different hyper parameters of model architectures.

Our model was first pretrained on the log *P* data set reported in a previous study, which covered a wide range of compound classes.^38^ The data set originally contained 14,050 compounds, and 13,889 were kept after exclusion of identified erroneous data points. Similar to the original study, 10% of them were randomly selected as an independent test set, while 20% of the remaining data points were included in the validation set. Data augmentation was conducted in the previous study by including tautomers,^38^ resulting in roughly a 10-time increase in the volume of the data set. Based on the augmented data set, we trained models with different combinations of hyperparameters, including the number of convolution layers, the dimension of embedding, the number of attention heads, and the learning rate during training. Best prediction performance for both the original validation set (i.e., only with original SMILES representation) and the augmented validation set (i.e., including tautomers) was achieved by the model with 3 convolution layers, 128 embedding dimensions, 4 attention heads, and 1 × 10^–4^ learning rate, as shown in Figure 2. To mitigate overfitting and improve generalization, each graph convolution layer was followed by a linear transformation and batch normalization, ensuring stable training dynamics. Dropout was applied at multiple stages, with a dropout rate of 0.1 for each graph convolution layer and 0.2 for multihead attention. Additionally, weight decay of 0.01 was used to prevent excessive parameter growth, and early stopping with a patience of 10 epochs was implemented to terminate training when validation performance stopped improving. Regarding the prediction performance on the independent test set, the best root-mean-square error (RMSE) scores for both the original test set and the augmented test set were 0.40 ± 0.02, and they were significantly better than the RMSE reported in the original study (i.e., 0.47 ± 0.02 for both the original and augmented test set).^38^ A comprehensive comparison with other models from previous studies,^38^ including associated neural networks, fragmental or atom-based methods, and quantum-chemistry-based calculations, is reported in SI Section S1.2. Given the fact that the experimental error of determining log *P* is in the range of 0.2–0.4 log units,^38^ the RMSE values of our model predictions are close to the best possible predictivity. It is also noticeable that models with a relatively large 256-dimensional embedding when trained with a relatively high learning rate of 1 × 10^–3^, experienced drastic deterioration in prediction performance. This phenomenon is consistent with previous findings that increasing the learning rate might deteriorate the generalization performance of complex DL models like the ones with 256-dimensional embedding in our case.^39^ Additionally, we noticed that the performance was slightly affected by the number of attention heads. With 3 attention heads, the model achieved RMSE scores of 0.41 ± 0.01 for the original test set and 0.42 ± 0.01 for the augmented test set. In contrast, with 5 attention heads, the scores were 0.40 ± 0.01 and 0.40 ± 0.02, respectively. After hyperparameter exploration with the training and validation log *P* data set, the model was trained on the whole log *P* data set with the above-mentioned best hyperparameter combination to prepare for the fine-tuning on the toxicity data set.

### Fine-Tuning for the Prediction of Toxicity Toward Nitrifiers

In the fine-tuning, the last two fully connected layers of the pretrained model were replaced with two newly initialized fully connected layers that functioned as a classifier. The objectives of fine-tuning were to retain the preacquired knowledge through subtle adjustments to the model weights and ensure a stable convergence. To this end, the model was fine-tuned with a smaller learning rate (i.e., 5 × 10^–5^). The performance of the fine-tuned model was evaluated through a rigorous stratified 10-fold cross-validation, ensuring the preservation of the positive-to-negative sample distribution in each fold. Furthermore, a model with the same architecture was trained from scratch only using the nitrification data set, eliminating the impact of the knowledge gained from the log *P* data set, thus serving as a baseline for comparison.

For comparative analysis, we developed several classic machine learning models using Molecular ACCess System (MACCS) fingerprints.^40^ This analysis included a range of models: Support Vector Machine (SVM),^41^ Random Forest (RF),^42^ Adaptive Boosting (AdaBoost),^43^ Extreme Gradient Boosting (XGBoost),^44^ K-Nearest Neighbor (KNN),^45^ and Multilayer Perceptron (MLP).^46^ We first assessed these models using a 10-fold cross-validation approach, testing various hyperparameter settings. Subsequently, the model was trained on the merged training and validation set using the hyperparameters that led to the best validation results before undergoing the final evaluation on the separate test data set. Details of the hyperparameter settings of each model for the 10-fold cross-validation as well as their validation accuracies can be found in SI Section S1.4. We also tested self-supervised models pretrained on millions of data, including ChemBERTa pretrained on 77 million molecules,^47^ MolFormer pretrained on 1.1 billion molecules,^48^ and MolCLR pretrained on 10 million molecules.^49^ They were fine-tuned with our curated toxicity data set. ChemBERTa and MolFormer were fine-tuned with the HuggingFace API,^50^ while MolCLR was fine-tuned within its original code.^49^

The comparison results are illustrated in Table 1. Notably, the fine-tuned graph model (i.e., FTGAT) demonstrated superior performance with a test ROC-AUC score of 0.85 ± 0.08 over the graph model (i.e., GAT) trained exclusively on the nitrification data set with a test ROC-AUC score of 0.82 ± 0.11, suggesting that our transfer learning indeed improved model performance. The classical machine learning models, namely, SVM, RF, AdaBoost, XGBoost, KNN, and MLP, showed test ROC-AUC scores ranging from 0.79 to 0.83. They thus performed worse than the fine-tuned graph model, confirming the superior effectiveness of graph-based neural network models with attention mechanisms in capturing the complex patterns in the molecular property data over traditional machine learning approaches. Among the models pretrained on large data sets, MolFormer showed the best test performance with a test ROC-AUC score of 0.85 ± 0.07. Notably, despite being pretrained on a substantially smaller data set, FTGAT achieved comparable test performance with MolFormer, indicating that pretraining with data mechanistically relevant to specific downstream tasks may enhance pretraining efficiency. Interestingly, the Graph Isomorphism Network (MolCLR_GIN) pretrained on 10 million molecules using a contrastive learning method demonstrated unexpectedly low performance (i.e., 0.71 ± 0.09). We hypothesized that the effectiveness of this pretraining approach might depend on the model architecture. Therefore, we pretrained a model (MolCLR_GAT) that shares the same architecture with GAT and FTGAT on the same 10 million molecules using contrastive learning, and it achieved a higher test ROC-AUC score of 0.83 ± 0.10. The detailed distribution of test ROC-AUC scores for each model can be found in SI Figure S7.

**Table 1:** Test Performance of Different Models^a^

no	model	mean ROC-AUC	standard deviation
1	SVM	0.79	0.10
2	RF	0.83	0.09
3	AdaBoost	0.80	0.10
4	XGBoost	0.83	0.07
5	KNN	0.80	0.12
6	MLP	0.81	0.09
7	ChemBERTa	0.79	0.05
8	MolFormer	**0.85**	0.07
9	MolCLR_GIN	0.71	0.09
10	GAT	0.82	0.11
11	MolCLR_GAT	0.83	0.10
12	FTGAT	**0.85**	0.08

a The first six models are supervised learning models, while models no. 7–9 are self-supervised/pre-trained models. The last three models have the same model architecture as proposed in this work: the first was not pre-trained, the second was pre-trained using the contrastive learning method,^49^ and the third was pre-trained with the log *P* dataset. The mean and standard deviation of the test ROC-AUC are reported. Models with the best performance are marked in bold.

Our model was further validated against *in vitro* experimental results obtained from external sources, as reported in two recent studies.^51,52^ The external data set consists of 10 compounds, including plant-derived chemicals and veterinary drugs. A rigorous check was conducted to prevent any overlap between the external data set and our curated nitrification data set. Following our predetermined criteria for positive and negative classification, albendazole was labeled as positive, while the remaining compounds were labeled as negative. Details of the compounds and prediction results can be found in SI Section S2.3. For this small external data set, our model achieved 100% prediction accuracy, with the positive compound, albendazole, attaining a high predicted probability of 0.97 of being toxic.

### Improving GNNs Interpretability with Multihead Attention and Shapley Values

**Figure 3:**
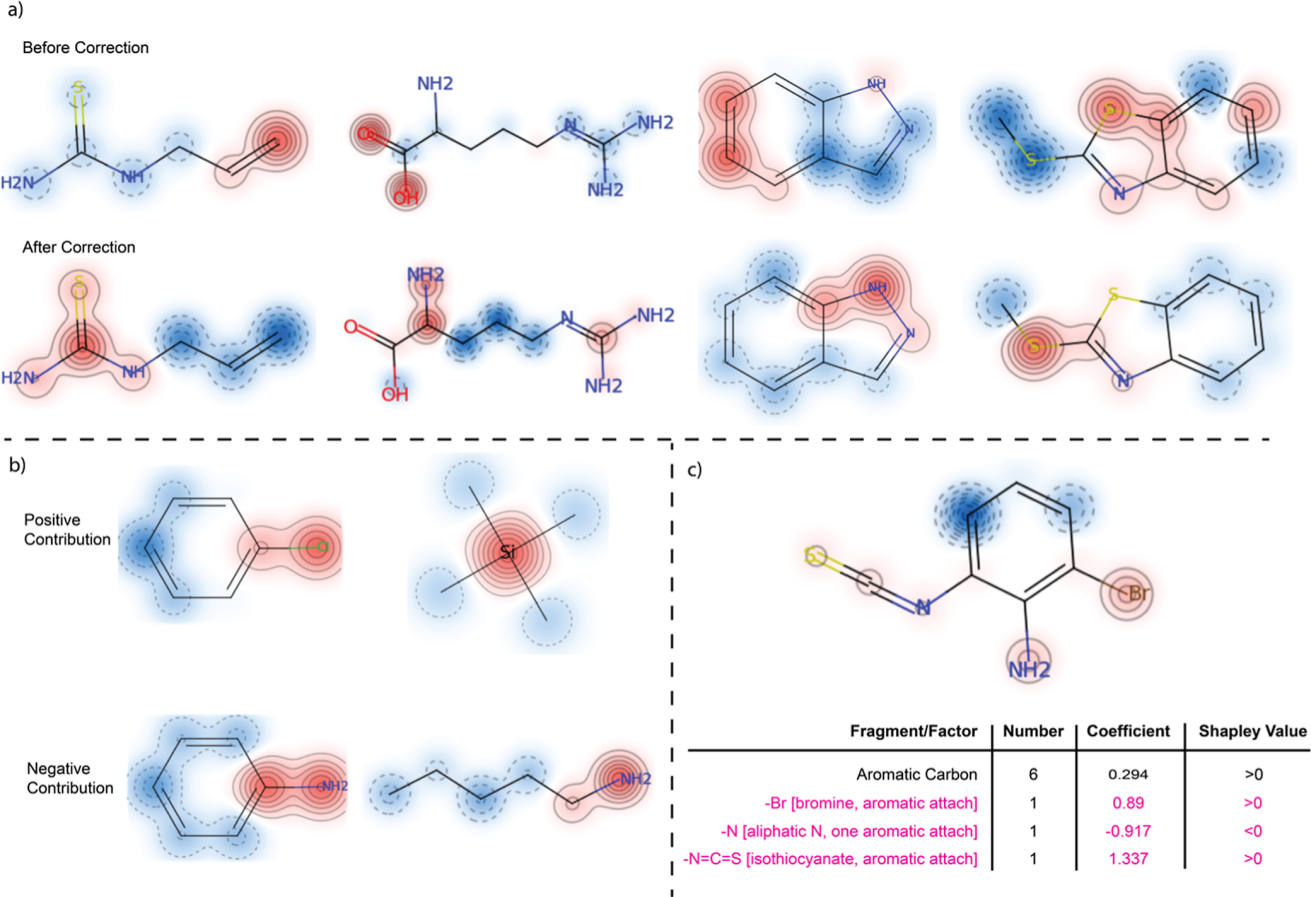
Visualization of attention mechanisms and their impact on log *P* prediction. (a) Comparison of attention distributions on molecular substructures before and after correction, with dark red indicating high attention and dark blue indicating low attention. Substructures with high attention weights are supposed to be important for decision-making. The first row illustrates attention allocation prior to correction, while the second row demonstrates adjusted attention after correction. (b) Influence of substructural attentions on log *P* values, where the first row contains compounds with aromatic chlorine and silyl groups that positively affect log *P*, and the second row shows compounds with aromatic and aliphatic amines negatively impacting log *P*. (c) Application of the attention-based method and Shapley values to determine the contributions of important substructures, compared with KOWWIN coefficients. Substructures (fragments/factors) with relatively large coefficients (absolute values) are highlighted.

Attention mechanisms have gained significant interest in the domain of computational chemistry, particularly for their role in the interpretability of DL models (e.g., GNNs) in predicting molecular properties.^53−55^ They can provide insights into which atomic or substructural features contribute most significantly to model predictions by focusing on relevant parts of molecular structures, which can potentially reveal the complex relationships between molecular structures and properties and also improve the prediction performance. In this study, we conducted a comparison between the model equipped with and without a multihead attention component, as illustrated in SI Figure S1. The inclusion of the multihead attention component substantially improved the predictive accuracy. For example, without multihead attention component, the RMSE scores for the original test set and augmented test set of the log *P* data set were increased from 0.40 ± 0.02 and 0.40 ± 0.02 to 0.42 ± 0.02 and 0.42 ± 0.02, suggesting a worse performance. However, the efficacy and reliability of attention mechanisms as interpretive tools are not always consistent.^56,57^ In certain scenarios, the attention weights may not align with chemically intuitive explanations. This discrepancy could be demonstrated in our investigation focused on the prediction of log *P* values since log *P* has been extensively examined and understood in terms of its relationship with chemical structures. Upon extracting and analyzing the attention weights derived from the multihead attention component of our model, as depicted in Figure 3a, it became apparent that despite achieving good predictive accuracy, the attention weights appeared to be distributed in a seemingly arbitrary manner, and they could not directly offer explanations that resonate with the established mechanistic understanding of log *P* values.

We posited that the failure of attention weights to highlight chemical structures relevant to model predictions can be attributed to the inherent dynamics of feature representation evolution with the GATConv layers. During the message-passing phase in the GATConv layers, the feature representation of each atom was updated by aggregating information from the immediate neighbors of that atom. After several convolutional layers, the feature representation of each atom was altered to encapsulate information about its connectivity up to a certain distance. For example, we employed four GATConv layers in the model, so the feature representation of an atom was an aggregation of information from all of the atoms within a four-bond reach. Consequently, the attention weights assigned in the multihead attention component postconvolution did not exclusively pertain to the original atom but to a united feature representation. To accurately assign the attention weights to the original atoms, we traced the convolution process and decomposed the united feature representations. Knowing the exact composition of the feature representation of each atom allowed us to correct the attention weights by distributing them to the original atoms, and the corrected attention weights were used for all of the subsequent analysis. The difference in attentions for log *P* prediction before and after corrections is shown in Figure 3a. After correction, the chemical structures with high corrected attention weights aligned well with the known structures that are critical to log *P*.

While attention weights were conventionally employed as indicators for the contributions of specific structures within the decision-making process of the model,^58,59^ this approach might not always yield accurate representations of contributions, especially in architectures where the multihead attention component is followed by layers such as fully connected networks. The complexity introduced by subsequent layers can obscure the direct relationship between attention weights and their actual influence on the model predictions. We provided simple examples in Figure 3b to demonstrate this issue. For instance, it is known that aromatic chloride increases log *P* while aromatic amine decreases log *P*, but they both obtained high attention weights in chlorobenzene and aniline, respectively. Therefore, Shapley values were applied in this study to more robustly quantify the contributions of identified substructures.

Our methodology for the computation of Shapley values was adapted from previous work.^26^ In the context of structure–activity relationship studies, the Shapley value is intended to be the average expected marginal contribution of a substructure across possible combinations with all the other atoms and bonds in the molecule.^26,60,61^ Yet, our model incorporated four GATConv layers, implying that the influence on a substructure was primarily caused by its immediate neighbors within a four-bond radius. To obtain both good approximation and computational efficiency, the marginal contribution of a substructure was calculated only with the atoms and bonds that can be reached by the substructure within four bonds. It is important to emphasize that during the process of calculating marginal contribution features of atoms or bonds that were excluded from the calculation, they were set to zeros rather than being removed from the molecular graph. This decision is grounded in the understanding that molecular graphs are very sensitive to alterations in their structures.^62^

By integrating our attention-correction method with Shapley values, we succeeded in efficiently identifying important chemical structures significantly related to model predictions and quantifying their contributions. This approach was validated against the KOWWIN program, an acronym for the Octanol–Water Partition Coefficient Program developed by the U.S. Environmental Protection Agency (EPA) within the framework of its EPI Suite (Estimation Programs Interface Suite).^63^ KOWWIN is designed to predict the log *P* of chemicals based on their molecular structures. Each substructure contributes to the overall log *P* value based on empirical data. The model coefficients assigned to each individual substructure in KOWWIN define whether the respective substructure contributes positively (e.g., coefficient >0) or negatively (e.g., coefficient <0) to the overall log *P* values. We highlighted those substructures, for selected compounds, with substantial impacts on the overall log *P* values, specifically those that possessed high absolute contribution values. We then investigated if these substructures also received high attention weights within our method and if their Shapley values were consistent with the contributions suggested by KOWWIN, as shown exemplarily in Figure 3c.

In our example, in Figure 3c, analysis identified three substructures with significant influence on the overall log *P* value according to KOWWIN: −Br, –N, and –N=C=S. The bromine and isothiocyanate substructure attached to the aromatic ring contributed positively, whereas the aromatic amine contributed negatively to the overall log *P* value. These substructures were also successfully detected by our attention-based method, and the Shapley values computed for these substructures aligned well with their contributions as in KOWWIN. For a broader validation, we selected a diverse set of seven compounds, with log *P* values ranging from −4.22 to 5.42, and compared our method with KOWWIN. Details of the comparison can be found in SI Section S4. Beyond this, we systematically quantified the similarity between our computed Shapley values and substructure contributions from KOWWIN. By applying kernel density estimation and calculating the area under the overlap of the two distributions, we showed in Figure S17 a strong similarity (0.8 on a scale of 0 to 1) between Shapley values and KOWWIN coefficients for 100 randomly selected compounds. These compounds had reliable log *P* predictions with their log *P* values predicted by the KOWWIN tool deviating no more than 20% from their experimental log *P* values.

### Analysis of Structural Alerts of Toxicity Toward Nitrifiers

**Figure 4:**
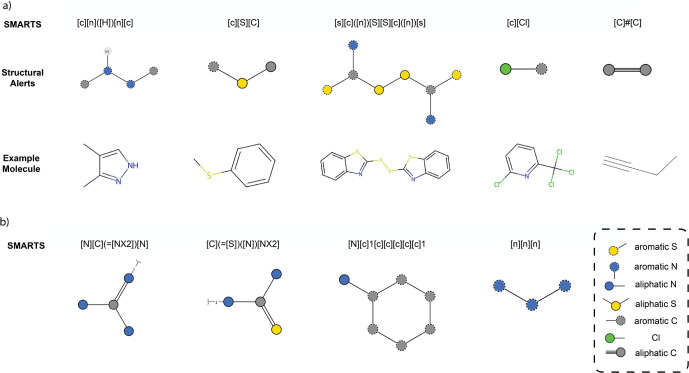
Structural alerts identified by the attention-based method and Shapley value. (a) Example of nitrogen-containing, sulfur-containing, nitrogen–sulfur containing, halogen-containing, and other structural alerts, along with an example molecule for each. (b) Examples of structural alerts with negative impacts on log *P* while contributing positively to toxicity.

Through an analysis leveraging our attention-based method and Shapley value assessments, we identified 24 substructures as structural alerts contributing positively to toxicity toward nitrifiers. These structural alerts were categorized into five distinct groups based on their atomic composition, focusing on elements such as nitrogen, sulfur, and halogens. Example structural alerts are shown in Figure 4a. A full list of these structural alerts and their example compounds are provided in SI Section S5.

The nitrogen-containing category involves seven structural alerts. The intrinsic presence of nitrogen indicates the potential for varied biological activities. It is hypothesized that chemicals containing these structural alerts might compete with ammonium for the active site of the AOM enzyme, thus inhibiting nitrification.^64−66^ The second category includes compounds with a sulfur-containing group and involves five structural alerts. Previous studies reported these structural alerts in potent nitrification inhibitors, and many of them are known metal-chelating compounds that inhibit enzymes like AOM, which require metals, particularly copper, for activation.^67,68^ The next category is the nitrogen–sulfur-containing group with seven structural alerts. This group offers potential insights into the complex interactions between the two elements and their combined effects on nitrification. For instance, thiourea, a structural alert in this category, is generally regarded as an extremely nonspecific ligand capable of forming complexes with various metals and acting as a potent scavenger of both hydroxy and superoxide radicals.^67,69^ In addition, we also identified a category of halogen-containing structural alerts, comprising three aromatic halogens, namely, aromatic chloride, aromatic bromide, and aromatic iodine. Halogen substituents on aromatic rings are electron-withdrawing and generally render the aromatic system more hydrophobic. The remaining structural alerts are the acetylenic and phenolic substructures. While acetylenic groups are known for their nitrification inhibition ability^70^ and also contribute to increased hydrophobicity, phenolic substructures are considered as general inhibitors of bacteria.^71,72^

It is commonly accepted that compounds with a higher log *P* value are more likely to act as baseline toxicants through increased intercalation into biological membranes and hence disruption of membrane functioning.^73^ Accordingly, statistically significant relationships between log *P* and EC50 values have also been demonstrated for bacteria, e.g., the inhibition of the luminescent bacterium *Vibrio fischeri*, often used in standard ecotoxicological testing.^14,74^ As has been shown for other membrane-bound enzymes, AMO, being a transmembrane enzyme, might be particularly vulnerable to disturbance of the bacterial membrane. It could therefore be speculated that structural alerts contributing to increasing log *P* would also positively contribute to nitrification toxicity. This hypothesis is supported by most structural alerts of nitrification toxicity also exhibiting positive Shapley values in the log *P* prediction model, which aligns with the observed improvements in predictive performance and robustness during fine-tuning. However, an exception is observed with nitrogen-containing structural alerts, including thiourea, aromatic amines, and certain triazines, which contribute negatively to log *P* but positively to toxicity, as detailed in SI Section S5.

This anomaly could be indicative of specific modes of action of compounds containing nitrogen-containing structural alerts. For the compounds that contain thiourea, allylthiourea is one of the best-known nitrification inhibitors. Its inhibitory effect is likely linked to the ability of thiourea to chelate copper ions.^75^ Previous studies indicated that AMO is a copper-centered enzyme, and copper is essential for its *in vivo* and *in vitro* activity, possibly facilitating the binding and activation of oxygen for the oxidation of ammonia.^21^ Experiments showed that the addition of copper to cell extracts of AOB resulted in a significant stimulation of nitrification.^76^ Furthermore, in the context of compounds containing aromatic amines (e.g., aniline), it was initially believed that they compete with ammonium for the active site of the AMO enzyme.^20^ Moreover, recent investigations revealed additional toxic effects of aniline that could potentially account for its toxicity toward nitrifiers. For example, aniline was shown to induce stress in cellular envelopes, impacting components including the cell membrane, periplasm, and peptidoglycan.^77^ Lastly, with regard to triazine compounds (e.g., simazine), their mode of action in inhibiting nitrification remains less explored. The example of nitrogen-containing structural alerts thus demonstrates that our strategy of pretraining on log *P* allows differentiating between molecular substructures that contribute to baseline toxicity and those that contribute to more specific modes of toxic action.

### Implications for Environmental Fate and (Eco-)toxicology Modeling

In this study, we developed a GNN consisting of a localized attention mechanism to effectively aggregate neighborhood feature information for atom-level message passing alongside a global attention mechanism to capture the global relationships and interactions within the molecule. The architecture of our model demonstrated proficient predictive performance across various regression and classification tasks, including solubility (i.e., ESOL),^33^ mutagenicity,^35^ hERG,^36^ and BBBP.^37^ Following pretraining on a log *P* data set and subsequent fine-tuning on a thoroughly curated data set for toxicity toward nitrifiers, the fine-tuned model surpassed classic machine learning models such as SVM, RF, AdaBoost, K-NN, and MLP in classification ROC-AUC scores and achieved similar or even better performance compared with self-supervised/pretrained models. It suggests that the strategic selection of pretraining data sets, aligned with the physiological or biochemical mechanisms under investigation, could optimize effective learning. Additionally, according to the learning curves in SI Figure S9, it showed a more consistent learning progression compared to its non-pretrained counterpart, with a steady reduction in training loss. Given the challenge of small data set sizes common to the field of environmental chemistry and (eco-)toxicology but also encountered in other scientific fields,^10^ our approach, by delving into toxicity toward nitrifiers as an illustrative example, serves as a proof of concept that mechanism-guided transfer learning from larger, relevant data sets can mitigate typical concerns such as overfitting and suboptimal predictive accuracy.

Moreover, this study enhanced the interpretability of global attention mechanisms by tracing and adjusting for the dynamic evolution of feature representations during message passing. This refinement led to a more successful identification of molecular substructures that are important for predictions. Traditional reliance on attention weights as proxies for the contribution of specific structures in the decision-making process of a model may not always reflect their true impacts, particularly in complex architectures where multihead attention is followed by fully connected layers, which can obscure the causal relationship between attention weights and prediction outcomes. To address this, we applied Shapley values specifically designed for graph-structured data for a more definitive quantification of the contributions of the identified important substructures. We enhanced the computational efficiency by using Shapley values for graph-structured data by analyzing them exclusively for substructures highlighted by the adjusted attention weights. For example, for the molecule chloronaphthalene, the calculation speed was accelerated by more than 70 times compared to calculating Shapley values for all possible substructures, reducing the calculation time from 29.01 to 0.4 s on a Mac mini (2018) with a 3.2 GHz 6-Core Intel Core i7 processor.

Finally, this work introduced a strategy for distinguishing between specific modes of action for toxicity toward nitrifiers and baseline toxicity by comparing structural alerts derived from nitrification toxicity classification and log *P* prediction models. This level of interpretability exceeds what can be achieved by using classical machine learning models. Structural alerts with positive contributions to toxicity but negative contributions to log *P* included thiourea, aromatic amines, and triazines, suggesting that they are responsible for unique modes of action other than baseline toxicity. Our strategy holds potential applicability in the modeling of (eco)toxicological effect end-points more broadly, such as in aquatic (eco-)toxicity. For the EnviroTox database—a repository of *in vivo* aquatic toxicity data—, for instance, it has been reported that only about 60% of chemicals could be confidently classified as baseline toxicants or assigned to other known modes of toxic action, such as reactive toxicity,^78^ neurotoxicity, oxidative phosphorylation uncoupling, or acetylcholinesterase inhibition.^79^ The remaining 40% of chemicals in commerce in the EnviroTox database could not be assigned to any known specific mode of toxic action, although, at least some of them seemed to be highly toxic. In this context, our suggested modeling strategy of graph-based transfer learning from log *P* as an indicator of baseline toxicity in combination with structural alert and Shapely value analysis can help uncover new classes of structural alerts. It could thus support major advances in mechanistic interpretation of available (eco-)toxicity data, which are critically needed for assessing the hazard and risk of chemicals in commerce but also for developing safer chemical alternatives and ultimately protecting the environment and human health from chemical risks. Altogether, our modeling strategy demonstrated here paves the way toward efficient transfer learning on small data sets in (eco-)toxicology, while providing mechanistically meaningful insights on structural alerts indicative of specific modes of toxic action.

## Data Availability

Log *P* data set reported in the previous publication^38^ were deposited at https://github.com/nadinulrich/log_P_prediction. Manually curated data set of toxicity toward nitrifiers, source Python code of our GNNs models, final model weights for logP and toxicity prediction, and demos of how to apply corrected global attention and Shapley values for XAI can be found at https://github.com/zhangky12/ToxPred_nitrification.

## Supporting Information Available

Comprehensive comparison of KOWWIN coefficients and Shapley values (XLSX)
Additional experimental details, model prediction results for extra data, 10-fold cross-validation results for toxicity tests, visualization of feature evolution during convolution, learning curves of transfer learning, details of the toxicity data set on nitrifiers, and full list of structural alerts (PDF)
