# Supplementary material for: Predicting Toxicity toward Nitrifiers by Attention-Enhanced Graph Neural Networks and Transfer Learning from Baseline Toxicity
Source: Environ Sci Technol. 2025 Feb 27;59(9):4518–29. doi: 10.1021/acs.est.4c12247 (PMC11912336; doi:10.1021/acs.est.4c12247)
Supplement: Supplementary file 2 — es4c12247_si_002.pdf [file es4c12247_si_002.pdf]

Predicting Toxicity towards Nitrifiers by Attention-Enhanced Graph Neural Networks and Transfer Learning from Baseline Toxicity

Kunyang Zhang[1,2,*], Philippe Schwaller[3,4], Kathrin Fenner[1,2]

[1]Department of Environmental Chemistry, Eawag, 8600 Dübendorf, Switzerland
[2]Department of Chemistry, University of Zürich, 8057 Zürich, Switzerland
[3]Laboratory of Artificial Chemical Intelligence (LIAC), Institute of Chemical Sciences and Engineering, EPFL, 1015 Lausanne, Switzerland
[4]National Centre of Competence in Research (NCCR) Catalysis, EPFL, 1015 Lausanne, Switzerland

This 37-page file includes:

Figures S1 to S17
Tables S1 to S10

Table of Contents

1. MODEL ARCHITECTURE AND PREDICTION PERFORMANCE **1**
1.1 MODEL WITHOUT MULTI-HEAD ATTENTION COMPONENT 1
1.2 LOGP PREDICTION PERFORMANCE COMPARSION 1
1.3 MODEL PREDICTION RESULTS FOR EXTRA DATA 1
1.4 10-FOLDS CROSS VALIDATION FOR TOXICITY TEST 2
1.5 FEATURE EVOLUTION IN CONVOLUTION 8
1.6 IMPACT OF TRANSFER LEARNING ON LEARNING CURVES 9
2. DATASET **9**
2.1 EXTRA DATASET 9
2.2 CURATED TOXICITY DATASET OF MICROBIAL NITRIFICATION 11
2.3 EXTERNAL *IN VITRO* EXPERIMENTAL DATA 26
3. GRAPH ATTENTION CONVOLUTION AND MULTI-HEAD GLOBAL ATTENTION **27**
4. KOWWIN COEFFICIENTS AND SHAPLEY VALUES **28**
5. STRUCTURAL ALERTS **33**
REFERENCES **37**

1. Model Architecture and Prediction Performance

1.1 Model Without Multi-Head Attention Component

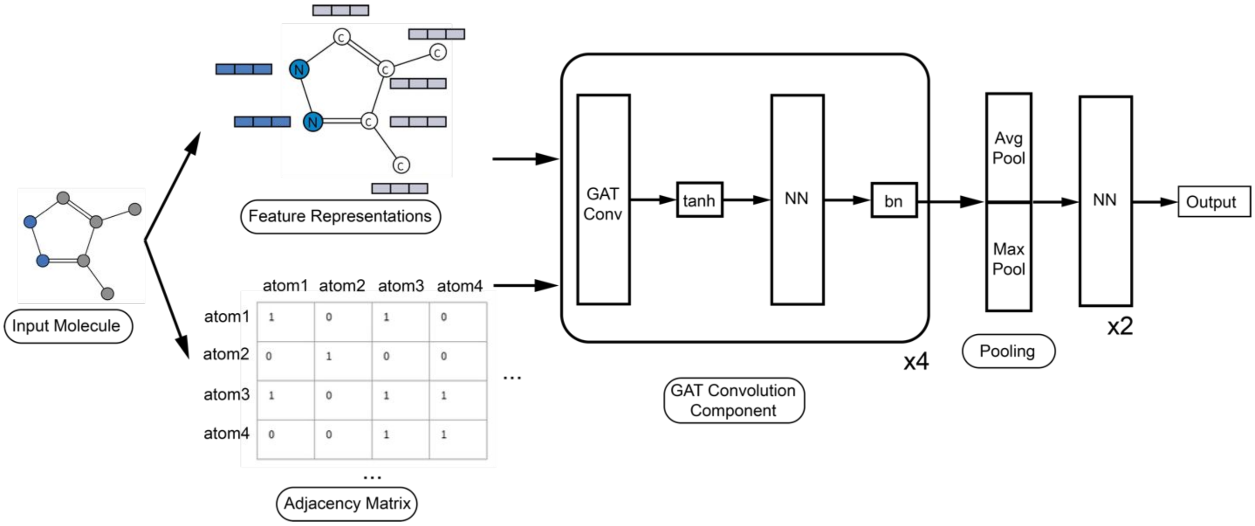

Figure S1. The architecture of model without global multi-head attention mechanisms. The input molecule is converted into feature representations and an adjacency matrix for convolution operations and two fully connected layers for final predictions.

1.2 LogP Prediction Performance Comparsion

Table S1: Comparison of the logP prediction performance.[1]

Model	Original SMILES	Augmented SMILES
DNN	0.47 ± 0.02	0.47 ± 0.02
ACD/GALAS	0.50 ± 0.03	0.65 ± 0.03
ALOGPS	0.50 ± 0.02	0.66 ± 0.03
COSMO-RS	0.97 ± 0.03	-
DataWarrior	0.80 ± 0.02	0.92 ± 0.02
JChem	0.72 ± 0.02	0.74 ± 0.03
KOWWIN	0.65 ± 0.04	0.92 ± 0.04
OCHEM	**0.34 ± 0.02**	0.65 ± 0.03
GAT	0.40 ± 0.02	**0.40 ± 0.02**

The root mean square error and corresponding variance for the logP prediction are given in the table. The model with best prediction performance for each set of SMILES is marked as bold. The results of the first 8 models are from the previous study.[1]

1.3 Model Prediction Results for Extra Data

Table S1: Prediction results for ESOL, Mutagenicity, hERG, and BBBP dataset.

Data	Task	Metric1	Performance1	Metric2	Performance2
ESOL	Regression	R^2	0.902 ± 0.03	RMSE	0.587 ± 0.02
Mutagenicity	Classification	ROC-AUC	0.914 ± 0.02	Accuracy	0.864 ± 0.03
hERG	Classification	ROC-AUC	0.947 ± 0.02	Accuracy	0.909 ± 0.02
BBBP	Classification	ROC-AUC	0.925 ± 0.04	Accuracy	0.892 ± 0.03

1.4 10-folds Cross Validation for Toxicity Test

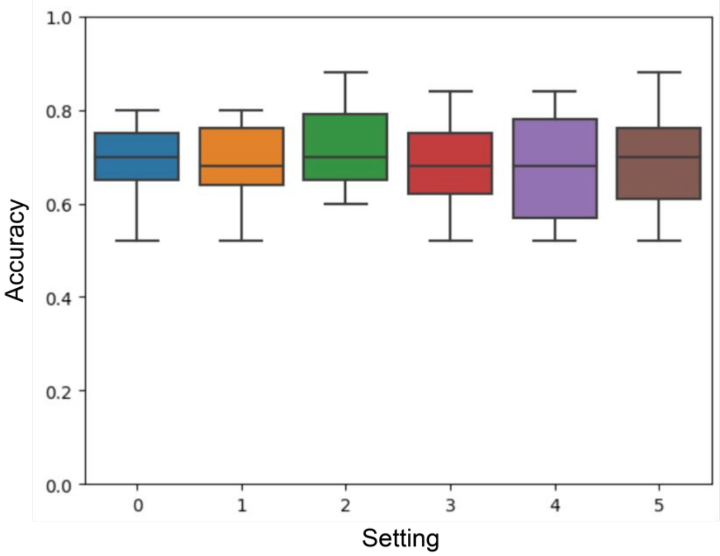

Setting

Model	C	kernel	gamma
0	1	rbf	scale
1	0.5	rbf	scale
2	2	rbf	scale
3	1	poly	scale
4	0.5	poly	scale
5	2	poly	scale

Figure S2. 10-folds cross validation results of SVM model and combinations of hyper-parameters.

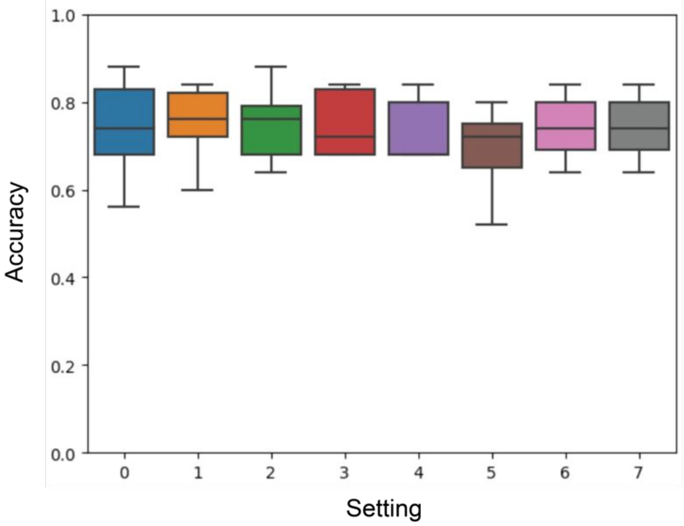

Setting

Model	n_estimators	criterion	min_samples_split
0	50	gini	2
1	100	gini	2
2	50	entropy	2
3	100	entropy	2
4	50	gini	4
5	100	gini	4
6	50	entropy	4
7	100	entropy	4

Figure S3. 10-folds cross validation results of RF model and combinations of hyper-parameters.

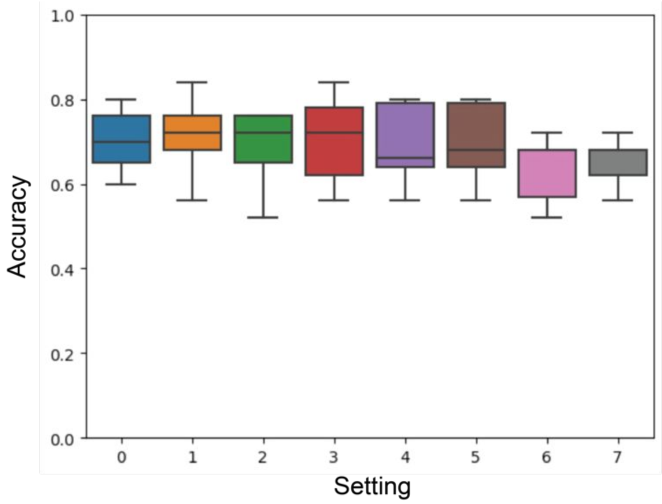

Setting

Model	n_estimators	learning_rate	algorithm
0	50	1	SAMME.R
1	100	1	SAMME.R
2	50	0.1	SAMME.R
3	100	0.1	SAMME.R
4	50	1	SAMME
5	100	1	SAMME
6	50	0.1	SAMME
7	100	0.1	SAMME

Figure S4. 10-folds cross validation results of AdaBoost model and combinations of hyper-parameters.

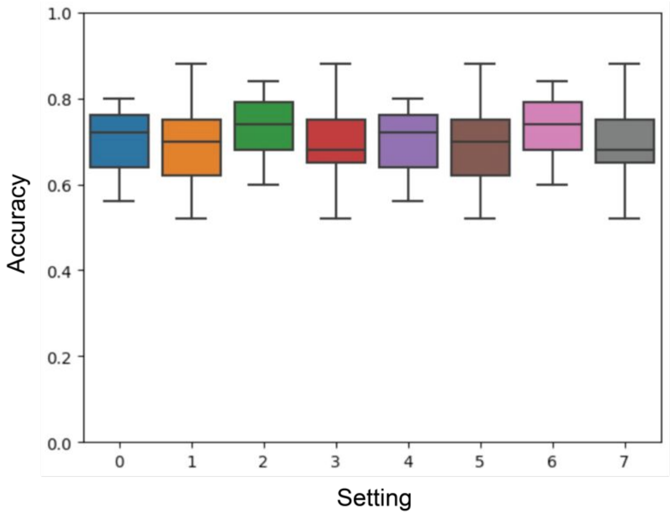

Setting

Model	n_neighbors	weights	algorithm
0	5	uniform	auto
1	3	uniform	auto
2	5	distance	auto
3	3	distance	auto
4	5	uniform	brute
5	3	uniform	brute
6	5	distance	brute
7	3	distance	brute

Figure S5. 10-folds cross validation results of KNN model and combinations of hyper-parameters.

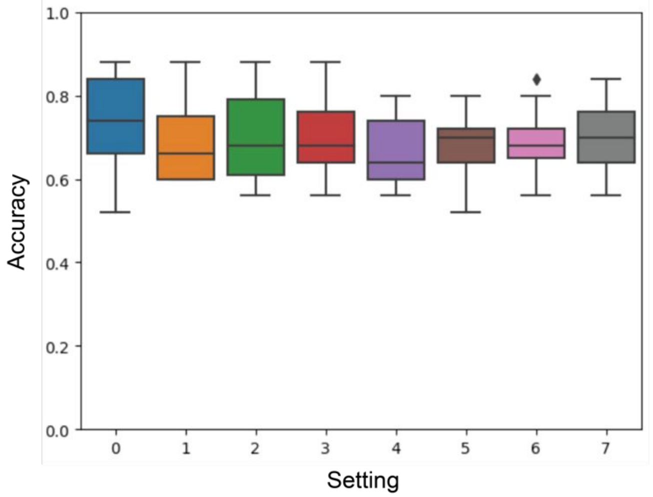

Setting

Model	hidden_layer_sizes	activation	solver
0	100	relu	adam
1	50	relu	adam
2	100	tanh	adam
3	50	tanh	adam
4	100	relu	sgd
5	50	relu	sgd
6	100	tanh	sgd
7	50	tanh	sgd

Figure S6. 10-folds cross validation results of MLP model and combinations of hyper-parameters.

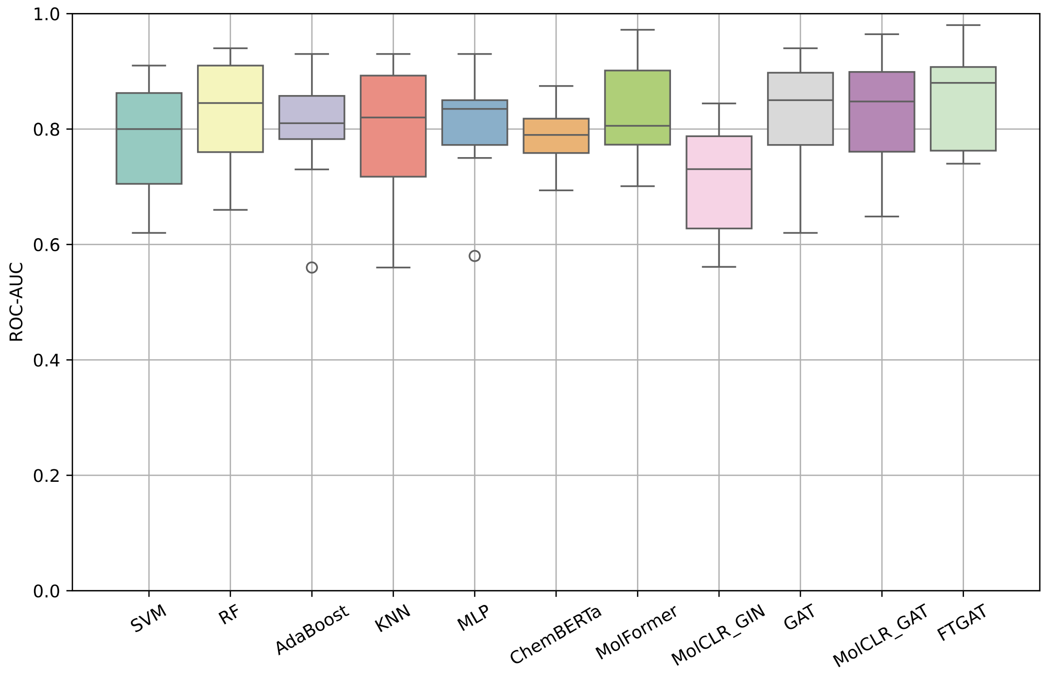

Figure S7. Comparison of test ROC-AUC scores on toxicity prediction with MACCS descriptors.

Table S2. Performance of random forest models using MACCS descriptors and additional hyperparameter tuning.

n_estimators	max_depth	class_weight	roc_auc_mean	std
50	3	balanced	0.751	0.071

50	3	balanced_subsample	0.768	0.085
50	5	balanced	0.785	0.102
50	5	balanced_subsample	0.8	0.077
50	10	balanced	0.827	0.099
50	10	balanced_subsample	0.831	0.095
50	20	balanced	**0.832**	0.092
50	20	balanced_subsample	0.823	0.101
50	None	balanced	0.823	0.103
50	None	balanced_subsample	0.828	0.108
100	3	balanced	0.759	0.066
100	3	balanced_subsample	0.766	0.079
100	5	balanced	0.8	0.075
100	5	balanced_subsample	0.789	0.077
100	10	balanced	0.827	0.09
100	10	balanced_subsample	0.832	0.095
100	20	balanced	0.821	0.096
100	20	balanced_subsample	0.831	0.099
100	None	balanced	0.831	0.103
100	None	balanced_subsample	0.823	0.091

Table S3. Performance of random forest models using Morgan descriptors (radius=2, fpSize=2048) and additional hyperparameter tuning.

**n_estimators**	**max_depth**	**class_weight**	**roc_auc_mean**	**std**
50	3	balanced	0.745	0.083
50	3	balanced_subsample	0.713	0.105
50	5	balanced	0.752	0.092
50	5	balanced_subsample	0.744	0.101
50	10	balanced	0.786	0.093
50	10	balanced_subsample	0.772	0.106
50	20	balanced	0.788	0.099
50	20	balanced_subsample	0.774	0.113
50	None	balanced	0.8	0.09
50	None	balanced_subsample	0.808	0.102
100	3	balanced	0.732	0.066
100	3	balanced_subsample	0.767	0.073
100	5	balanced	0.78	0.073
100	5	balanced_subsample	0.754	0.098
100	10	balanced	0.783	0.108

100	10	balanced_subsample	0.786	0.084
100	20	balanced	0.799	0.084
100	20	balanced_subsample	0.792	0.098
100	None	balanced	**0.814**	0.088
100	None	balanced_subsample	0.799	0.103

Table S4. Performance of XGBoost models using MACCS descriptors.

booster	max_depth	subsample	roc_auc_mean	std
gbtree	3	0.6	0.826	0.104
gbtree	3	0.7	0.833	0.083
gbtree	3	0.8	0.827	0.087
gbtree	3	1	0.833	0.068
gbtree	5	0.6	0.82	0.088
gbtree	5	0.7	0.827	0.089
gbtree	5	0.8	0.831	0.077
gbtree	5	1	**0.834**	0.086
gbtree	10	0.6	0.822	0.097
gbtree	10	0.7	0.832	0.083
gbtree	10	0.8	0.82	0.078
gbtree	10	1	0.829	0.077
gbtree	20	0.6	0.819	0.092
gbtree	20	0.7	0.832	0.075
gbtree	20	0.8	0.828	0.081
gbtree	20	1	**0.834**	0.071
gbtree	None	0.6	0.832	0.088
gbtree	None	0.7	0.824	0.089
gbtree	None	0.8	0.829	0.081
gbtree	None	1	0.819	0.085
dart	3	0.6	0.828	0.103
dart	3	0.7	0.816	0.086
dart	3	0.8	0.822	0.102
dart	3	1	0.833	0.068
dart	5	0.6	0.813	0.097
dart	5	0.7	0.823	0.089
dart	5	0.8	0.829	0.083
dart	5	1	**0.834**	0.086
dart	10	0.6	0.824	0.091
dart	10	0.7	0.806	0.069

dart	10	0.8	0.816	0.079
dart	10	1	0.819	0.077
dart	20	0.6	0.825	0.086
dart	20	0.7	0.833	0.084
dart	20	0.8	0.822	0.088
dart	20	1	**0.834**	0.071
dart	None	0.6	0.826	0.098
dart	None	0.7	0.825	0.094
dart	None	0.8	0.833	0.078
dart	None	1	0.819	0.085
gblinear	None	None	0.758	0.101

Table S5. Performance of XGBoost models using Morgan descriptors (radius=2, fpSize=2048).

**booster**	**max_depth**	**subsample**	**roc_auc_mean**	**std**
gbtree	3	0.6	0.765	0.095
gbtree	3	0.7	0.778	0.072
gbtree	3	0.8	0.775	0.1
gbtree	3	1	0.765	0.109
gbtree	5	0.6	0.778	0.087
gbtree	5	0.7	0.779	0.087
gbtree	5	0.8	0.789	0.086
gbtree	5	1	0.791	0.094
gbtree	10	0.6	0.775	0.106
gbtree	10	0.7	0.761	0.076
gbtree	10	0.8	0.793	0.087
gbtree	10	1	0.793	0.085
gbtree	20	0.6	0.778	0.096
gbtree	20	0.7	0.761	0.07
gbtree	20	0.8	0.787	0.082
gbtree	20	1	0.783	0.097
gbtree	None	0.6	0.772	0.091
gbtree	None	0.7	0.787	0.072
gbtree	None	0.8	0.784	0.093
gbtree	None	1	0.787	0.086
dart	3	0.6	0.761	0.102
dart	3	0.7	0.767	0.095
dart	3	0.8	0.774	0.104
dart	3	1	0.765	0.109

dart	5	0.6	0.777	0.087
dart	5	0.7	0.793	0.108
dart	5	0.8	0.783	0.09
dart	5	1	0.791	0.094
dart	10	0.6	0.777	0.097
dart	10	0.7	0.784	0.083
dart	10	0.8	**0.795**	0.086
dart	10	1	0.793	0.085
dart	20	0.6	0.768	0.095
dart	20	0.7	0.768	0.077
dart	20	0.8	0.781	0.085
dart	20	1	0.783	0.097
dart	None	0.6	0.773	0.094
dart	None	0.7	0.78	0.092
dart	None	0.8	0.784	0.095
dart	None	1	0.787	0.086
gblinear	None	None	0.718	0.085

1.5 Feature Evolution in Convolution

Initial Composition

	atom0	atom1	atom2	atom3	atom4	atom5	atom6
atom0	1.0000	0.0000	0.0000	0.0000	0.0000	0.0000	0.0000
atom1	0.0000	1.0000	0.0000	0.0000	0.0000	0.0000	0.0000
atom2	0.0000	0.0000	1.0000	0.0000	0.0000	0.0000	0.0000
atom3	0.0000	0.0000	0.0000	1.0000	0.0000	0.0000	0.0000
atom4	0.0000	0.0000	0.0000	0.0000	1.0000	0.0000	0.0000
atom5	0.0000	0.0000	0.0000	0.0000	0.0000	1.0000	0.0000
atom6	0.0000	0.0000	0.0000	0.0000	0.0000	0.0000	1.0000

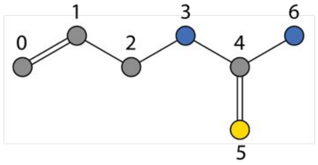

Composition after 1st convolution (C_1)

	atom0	atom1	atom2	atom3	atom4	atom5	atom6
atom0	0.5161	0.4839	0.0000	0.0000	0.0000	0.0000	0.0000
atom1	0.3305	0.3060	0.3635	0.0000	0.0000	0.0000	0.0000
atom2	0.0000	0.3254	0.2773	0.3974	0.0000	0.0000	0.0000
atom3	0.0000	0.0000	0.2903	0.4032	0.3065	0.0000	0.0000
atom4	0.0000	0.0000	0.0000	0.2770	0.1811	0.2388	0.3031
atom5	0.0000	0.0000	0.0000	0.0000	0.4272	0.5728	0.0000
atom6	0.0000	0.0000	0.0000	0.0000	0.4143	0.0000	0.5857

Composition after 2nd convolution (C_2 X C_1)

	atom0	atom1	atom2	atom3	atom4	atom5	atom6
atom0	0.4271	0.3987	0.1742	0.0000	0.0000	0.0000	0.0000
atom1	0.2472	0.3706	0.2115	0.1707	0.0000	0.0000	0.0000
atom2	0.1503	0.1932	0.3214	0.2188	0.1162	0.0000	0.0000
atom3	0.0000	0.0652	0.1111	0.3253	0.1688	0.1453	0.1844
atom4	0.0000	0.0000	0.0286	0.0968	0.3616	0.3134	0.1996
atom5	0.0000	0.0000	0.0000	0.1257	0.3155	0.4211	0.1376
atom6	0.0000	0.0000	0.0000	0.1692	0.2719	0.1458	0.4131

Composition after 3rd convolution (C_3 X C_2 X C_1)

	atom0	atom1	atom2	atom3	atom4	atom5	atom6
atom0	0.3156	0.3813	0.1973	0.1058	0.0000	0.0000	0.0000
atom1	0.2474	0.2858	0.2584	0.1477	0.0607	0.0000	0.0000
atom2	0.0684	0.1479	0.1435	0.2821	0.1237	0.1033	0.1311
atom3	0.0091	0.0356	0.0766	0.1878	0.2762	0.2328	0.1819
atom4	0.0000	0.0092	0.0231	0.1633	0.2898	0.2486	0.2660
atom5	0.0000	0.0000	0.0190	0.1065	0.3462	0.3494	0.1789
atom6	0.0000	0.0000	0.0130	0.1364	0.3125	0.2218	0.3163

Composition after 4th convolution (C_4 X C_3 X C_2 X C_1)

	atom0	atom1	atom2	atom3	atom4	atom5	atom6
atom0	0.2879	0.3425	0.2221	0.1228	0.0247	0.0000	0.0000
atom1	0.1700	0.2384	0.1787	0.2085	0.0775	0.0559	0.0710
atom2	0.0880	0.1264	0.1402	0.1912	0.1890	0.1443	0.1208
atom3	0.0135	0.0388	0.0570	0.1891	0.2592	0.2208	0.2214
atom4	0.0020	0.0112	0.0319	0.1518	0.3027	0.2582	0.2422
atom5	0.0000	0.0063	0.0218	0.1455	0.3075	0.2802	0.2387
atom6	0.0000	0.0056	0.0192	0.1528	0.2986	0.2382	0.2855

Figure S8. Feature evolution during convolution. Features of each atom evolve as they incorporate information from neighboring atoms. Values indicate the contribution from different atoms. With each convolution layer, the central atom's feature vector updates, reflecting the influence of adjacent atoms. For example, atom0 initially only has the contribution from itself, but eventually contains the contribution from atom4 after four convolutions.

1.6 Impact of Transfer Learning on Learning Curves

The training and test curves for the fine-tuned model are smoother than those of the model directly trained on the toxicity towards nitrification dataset.

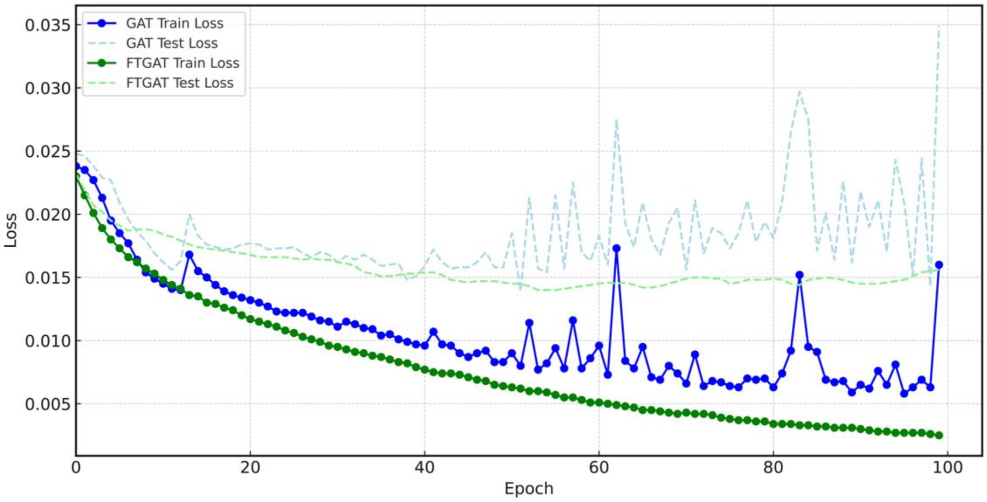

Figure S9. The learning curves of fine-tuned (i.e., FTGAT) and not fine-tuned (i.e., GAT) models.

2. Dataset

2.1 Extra Dataset

ESOL Dataset

The solubility dataset within MoleculeNet, known as ESOL, contains water solubility data for 1128 compounds.[2] This dataset is utilized to train models that predict aqueous solubility, a crucial parameter in various scientific and industrial applications such as drug development and environmental science. 113 compounds were randomly selected for testing while the remaining compounds were included in model training.

Mutagenicity Dataset

The Mutagenicity dataset from the Toxicity Benchmark database comprises 6512 chemicals annotated for Ames mutagenicity, presenting a binary classification challenge.[3] Aimed at advancing in silico prediction methodologies, this dataset serves as a critical benchmark for

evaluating the efficacy of computational models in determining chemical compounds' potential to induce genetic mutations. It's a valuable resource for researchers focused on chemical safety and environmental health sciences, facilitating improvements in predictive toxicology. 652 compounds were randomly selected for testing while the remaining compounds were included in model training.

hERG Dataset

The hERG dataset, as curated from ChEMBL, stands as a pivotal resource for the study of cardiac toxicity in the realm of drug discovery and safety assessment.[4] Comprising 10572 compounds, this dataset is characterized by its detailed representation of molecular structures, with an average of 29.39 nodes and 94.09 edges per graph. Its significance lies in the support it offers for the development of predictive models concerning the hERG potassium ion channel, which is crucial due to the channel's association with the risk of cardiac arrhythmias. By providing a robust foundation for computational models to predict the potential blockade of the hERG channel by small molecules, the dataset contributes to the advancement of safer pharmaceutical agents. Researchers utilize this dataset to explore the intricate balance between therapeutic efficacy and the mitigation of cardiac safety risks, thereby enhancing the drug development pipeline's efficiency and safety profile. 1058 compounds were randomly selected for testing while the remaining compounds were included in model training.

BBBP Dataset

The Blood-Brain Barrier Penetration (BBBP) dataset, curated as part of the MoleculeNet benchmarking dataset, is a resource for the prediction and study of permeability through the blood-brain barrier (BBB).[5] This dataset comprises 2039 molecular compounds, each labeled with binary indicators to signify their capability to penetrate the BBB—a critical consideration in the development of neurologically active therapeutics. 204 compounds were randomly selected for testing while the remaining compounds were included in model training.

2.2 Curated Toxicity Dataset of Microbial Nitrification

Name	Structure of Smiles	Smiles	Activity	Reference
Acetone	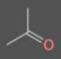	CC(=O)C	n	Hooper et al., 1973
Allythiourea	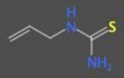	C=CCNC(=S)N	p	Hooper et al., 1973
Aminoethanol	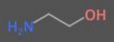	C(CO)N	n	Hooper et al., 1973
Aminoguanidine	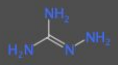	C(=NN)(N)N	p	Hooper et al., 1973
3-Aminotriazole	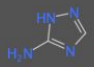	C1=NNC(=N1)N	p	Hooper et al., 1973
L-Arginine	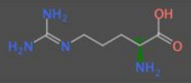	C(CC(C(=O)O)N)CN=C(N)N	p	Clark et al., 1967
N-Butanol	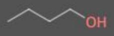	CCCCO	n	Hooper et al., 1973
2-Chloro-6-trichloro-methyl-pyridine	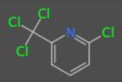	ClC1=CC=CC(=N1)C(Cl)(Cl)Cl	p	Hooper et al., 1973
Dichlorophenolinde-phenol	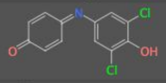	C1=CC(=O)C=CC1=NC2=CC(=C(C(=C2)Cl)O)Cl	n	Hooper et al., 1973
Dicyclohexylcarbo-diimide	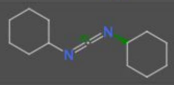	C1CCC(CC1)N=C=NC2CCCCC2	p	Hooper et al., 1973
Diethyldithiocarbamate	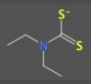	CCN(CC)C(=S)[S-]	p	Hooper et al., 1973
2,4-Dinitrophenol	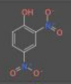	C1=CC(=C(C=C1[N+](=O)[O-])[N+](=O)[O-])O	n	Hooper et al., 1973
Diphenylthiocarbazone	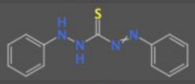	C1=CC=C(C=C1)NNC(=S)N=NC2=CC=CC=C2	p	Hooper et al., 1973
Dipyridyl	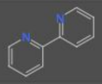	C1=CC=NC(=C1)C2=CC=CC=N2	p	Hooper et al., 1973
Ethanol	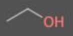	CCO	n	Hooper et al., 1973
Ethyl acetate	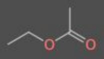	CCOC(=O)C	n	Hooper et al., 1973
Ethyl xanthate	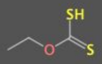	CCOC(=S)S	p	Hooper et al., 1973
L-Histidine	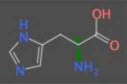	C1=C(NC=N1)CC(C(=O)O)N	p	Clark et al., 1967
L-Lysine	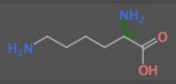	C(CCN)CC(C(=O)O)N	p	Clark et al., 1967
L-Methionine	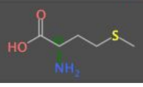	CSCCC(C(=O)O)N	p	Clark et al., 1967

Name	Structure of Smiles	Smiles	Activity	Reference
Nitrourea	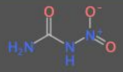	C(=O)(N)N[N+](=O)[O-]	n	Lees et al., 1957
o-Phenanthroline	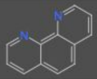	C1=CC2=C(C3=C(C=CC=N3)C=C2)N=C1	p	Hooper et al., 1973
Phenazine methosulfate	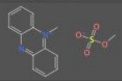	C[N+]1=C2C=CC=CC2=NC3=CC=CC=C31.COS(=O)(=O)[O-]	p	Hooper et al., 1973
N-Propanol	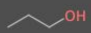	CCCO	n	Hooper et al., 1973
Quinacrine	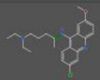	CCN(CC)CCCC(C)NC1=C2C=C(C=CC2=NC3=C1C=CC(=C3)Cl)OC	n	Zavarzin et al., 1958
Tetramethylammonium chloride	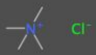	C[N+](C)(C)C.[Cl-]	n	Lees et al., 1946
Thiosemicarbazide	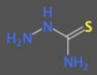	C(=S)(N)NN	p	Hooper et al., 1973
L-Threonine	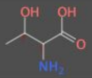	CC(C(C(=O)O)N)O	p	Clark et al., 1967
Trimethylamine	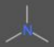	CN(C)C	n	Lees et al., 1946
L-Valine	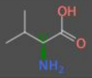	CC(C)C(C(=O)O)N	p	Clark et al., 1967
Allyl alcohol	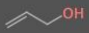	C=CCO	p	Tomlinson et al., 1966
Allyl chloride	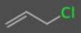	C=CCCl	n	Tomlinson et al., 1966
Allyl isothiocyanate	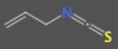	C=CCN=C=S	p	Tomlinson et al., 1966
Benzothiazole disulfide	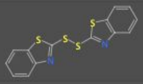	C1=CC=C2C(=C1)N=C(S2)SSC3=NC4=CC=CC=C4S3	p	Tomlinson et al., 1966
Carbon disulfide	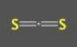	C(=S)=S	p	Tomlinson et al., 1966
Chloroform	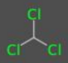	C(Cl)(Cl)Cl	p	Tomlinson et al., 1966
o-Cresol	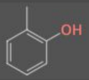	CC1=CC=CC=C1O	p	Tomlinson et al., 1966
Di-allyl ether	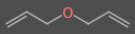	C=CCOCC=C	n	Tomlinson et al., 1966
Dicyandiamide	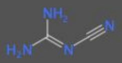	C(#N)N=C(N)N	n	Tomlinson et al., 1966
Diguanide	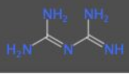	C(=NC(=N)N)(N)N	p	Tomlinson et al., 1966

Name	Structure of Smiles	Smiles	Activity	Reference
Dithio-oxamide	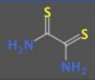	C(=S)(C(=S)N)N	p	Tomlinson et al., 1966
Guanidine carbonate	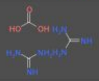	C(=N)(N)N.C(=N)(N)N.C(=O)(O)O	p	Tomlinson et al., 1966
8-Hydroxyquinoline	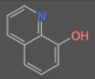	C1=CC2=C(C(=C1)O)N=CC=C2	n	Tomlinson et al., 1966
Mercaptobenzothiazole	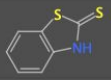	C1=CC=C2C(=C1)NC(=S)S2	p	Tomlinson et al., 1966
Methyl isothiocyanate	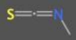	CN=C=S	p	Tomlinson et al., 1966
Methyl thiuronium sulfate	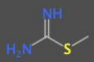	CSC(=N)N	p	Tomlinson et al., 1966
Phenol	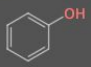	C1=CC=C(C=C1)O	p	Tomlinson et al., 1966
Potassium thiocyanate	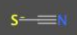	C(#N)[S-]	p	Tomlinson et al., 1966
Skatol	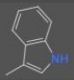	CC1=CNC2=CC=CC=C12	p	Tomlinson et al., 1966
Sodium dimethyl dithiocarbamate	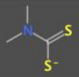	CN(C)C(=S)[S-]	p	Tomlinson et al., 1966
Sodium methyl dithiocarbamate	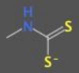	CNC(=S)[S-]	p	Tomlinson et al., 1966
Tetramethyl thiuram disulfide	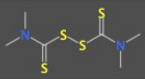	CN(C)C(=S)SSC(=S)N(C)C	p	Tomlinson et al., 1966
Thioacetamide	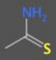	CC(=S)N	p	Tomlinson et al., 1966
Thiourea	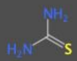	C(=S)(N)N	p	Tomlinson et al., 1966
Dodecylamine	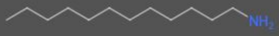	CCCCCCCCCCCCN	n	Hockenbury et al., 1977
Aniline	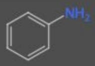	C1=CC=C(C=C1)N	p	Hockenbury et al., 1977
n-Methylaniline	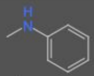	CNC1=CC=CC=C1	p	Hockenbury et al., 1977
1-Naphthylamine	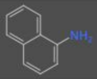	C1=CC=C2C(=C1)C=CC=C2N	p	Hockenbury et al., 1977
Ethylenediamine	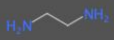	C(CN)N	p	Hockenbury et al., 1977
Napthylethylenediamine	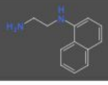	C1=CC=C2C(=C1)C=CC=C2NCCN	p	Hockenbury et al., 1977

Name	Structure of Smiles	Smiles	Activity	Reference
p-Nitroaniline	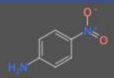	C1=CC(=CC=C1N)[N+](=O)[O-]	p	Hockenbury et al., 1977
p-Aminopropiophenone	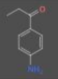	CCC(=O)C1=CC=C(C=C1)N	p	Hockenbury et al., 1977
Benzidine diHCl	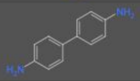	C1=CC(=CC=C1C2=CC=C(C=C2)N)N	p	Hockenbury et al., 1977
p-Phenylazoaniline	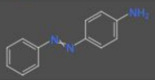	C1=CC=C(C=C1)N=NC2=CC=C(C=C2)N	n	Hockenbury et al., 1977
Hezamethylene diamine	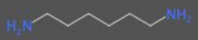	C(CCCN)CCN	n	Hockenbury et al., 1977
p-Nitrobenzaldehyde	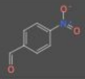	C1=CC(=CC=C1C=O)[N+](=O)[O-]	n	Hockenbury et al., 1977
Triethylamine	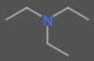	CCN(CC)CC	n	Hockenbury et al., 1977
Ninhydrin	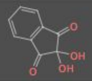	C1=CC=C2C(=C1)C(=O)C(C2=O)(O)O	n	Hockenbury et al., 1977
Benzocaine	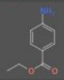	CCOC(=O)C1=CC=C(C=C1)N	n	Hockenbury et al., 1977
Dimethylgloxime	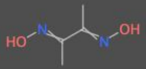	CC(=NO)C(=NO)C	n	Hockenbury et al., 1977
Benzylamine	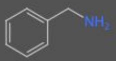	C1=CC=C(C=C1)CN	n	Hockenbury et al., 1977
Acetamide	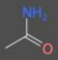	CC(=O)N	n	Hockenbury et al., 1977
Acetonitrile	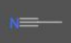	CC#N	n	Hockenbury et al., 1977
Acrylonitrile	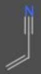	C=CC#N	n	Hockenbury et al., 1977
p-Aminobenzoic acid	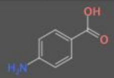	C1=CC(=CC=C1C(=O)O)N	n	Hockenbury et al., 1977
2-Amino-3,5-diiodobenzoic acid	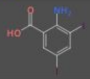	C1=C(C=C(C(=C1C(=O)O)N)I)I	p	Hockenbury et al., 1977
1-Amino-2-naphthol-4-sulfonic acid	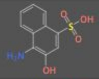	C1=CC=C2C(=C1)C(=CC(=C2N)O)S(=O)(=O)O	n	Hockenbury et al., 1977
Anthranilic acid	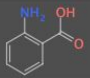	C1=CC=C(C(=C1)C(=O)O)N	n	Hockenbury et al., 1977
Azobenzene	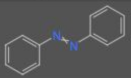	C1=CC=C(C=C1)N=NC2=CC=CC=C2	n	Hockenbury et al., 1977
Brucine	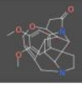	COC1=C(C=C2C(=C1)C34CCN5C3CC6C7C4N2C(=O)CC7OCC=C6C5)OC	n	Hockenbury et al., 1977

Name	Structure of Smiles	Smiles	Activity	Reference
sec-Butylamine	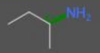	CCC(C)N	n	Hockenbury et al., 1977
Chlorobenzene	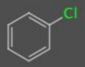	C1=CC=C(C=C1)Cl	n	Hockenbury et al., 1977
1-Chloro-4-nitrobenzene	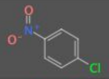	C1=CC(=CC=C1[N+](=O)[O-])Cl	n	Hockenbury et al., 1977
Citric acid	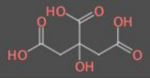	C(C(=O)O)C(CC(=O)O)(C(=O)O)O	n	Hockenbury et al., 1977
Diethanolamine	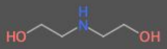	C(CO)NCCO	n	Hockenbury et al., 1977
Diethylamine	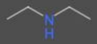	CCNCC	n	Hockenbury et al., 1977
Dimethylamine hydrochloride	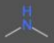	CNC	n	Hockenbury et al., 1977
Dimethylaminoazobenzene	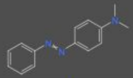	CN(C)C1=CC=C(C=C1)N=NC2=CC=CC=C2	n	Hockenbury et al., 1977
Diphenylamine	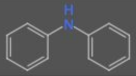	C1=CC=C(C=C1)NC2=CC=CC=C2	n	Hockenbury et al., 1977
p-Diphenylamine-sulfuric acid	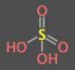	OS(O)(=O)=O	n	Hockenbury et al., 1977
Diphenylcarbazone	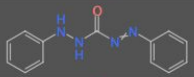	C1=CC=C(C=C1)NNC(=O)N=NC2=CC=CC=C2	n	Hockenbury et al., 1977
1,5-Diphenylcarbohydrazide	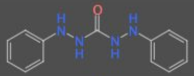	C1=CC=C(C=C1)NNC(=O)NNC2=CC=CC=C2	n	Hockenbury et al., 1977
Hezamethylenetetramine	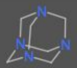	C1N2CN3CN1CN(C2)C3	n	Hockenbury et al., 1977
Melamine	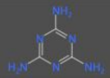	C1(=NC(=NC(=N1)N)N)N	n	Hockenbury et al., 1977
Propylamine	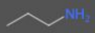	CCCN	n	Hockenbury et al., 1977
Rhodamine B	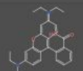	CCN(CC)C1=CC2=C(C=C1)C(=C3C=CC(=[N+](CC)CC)C=C3O2)C4=CC=CC=C4C(=O)O	n	Hockenbury et al., 1977
Sodium citrate	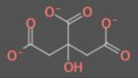	C(C(=O)[O-])C(CC(=O)[O-])(C(=O)[O-])O	n	Hockenbury et al., 1977
Stearic acid	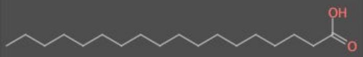	CCCCCCCCCCCCCCCCCC(=O)O	n	Hockenbury et al., 1977
Sulfamic acid	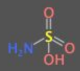	NS(=O)(=O)O	n	Hockenbury et al., 1977
Sulfanilamide	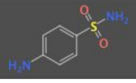	C1=CC(=CC=C1N)S(=O)(=O)N	n	Hockenbury et al., 1977

Name	Structure of Smiles	Smiles	Activity	Reference
Tributylamine	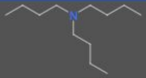	CCCCN(CCCC)CCCC	n	Hockenbury et al., 1977
Triethanolamine	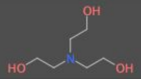	C(CO)N(CCO)CCO	n	Hockenbury et al., 1977
DMPP (3, 4-Dimethylpyrazol-phosphate)	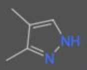	CC1=NNC=C1C	p	Prasad and Power, 1995
AM (2-amino-4-chloro-6-methylpyrimidine	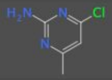	CC1=CC(=NC(=N1)N)Cl	p	Ranney, 1978
ATC (4-amino-1,2,4,-triazole hydrochloride)	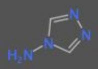	C1=NN=CN1N	p	Guthrie and Bomke, 1980
MT (3 mercapto-1,2,4-triazole)	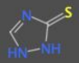	C1=NC(=S)NN1	p	Subbarao et al., 2005
MAST (2-amino-4-methyl-6-trichloromethyltriazine)	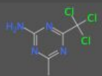	CC1=NC(=NC(=N1)N)C(Cl)(Cl)Cl	p	Subbarao et al., 2005
ST (2-sulfanilamidothiazole)	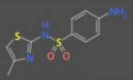	CC1=CSC(=N1)NS(=O)(=O)C2=CC=C(C=C2)N	p	Mitsui Toatsu, 1968
DCS (N-2,5,-dichlorophenyl succinamide)	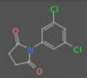	C1CC(=O)N(C1=O)C2=CC(=CC(=C2)Cl)Cl	p	Mosier et al., 1996
MPC (3-methyl-pyrazole-1-carboxamide)	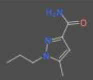	CCCN1C(=CC(=N1)C(=O)N)C	p	McCarty and Bremner, 1989
CP (2-cyanimino-4-hydroxy-6-methylpyrimidine)	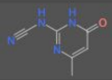	CC1=CC(=O)NC(=N1)NC#N	p	Subbarao et al., 2005
ATS (Ammonium thiosulfate)	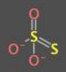	[O-]S([O-])(=O)=S	p	Goos, 1985
Dwell (etridiazole)	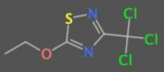	CCOC1=NC(=NS1)C(Cl)(Cl)Cl	p	Varsa et al., 1981
Sodium thiocarbonate	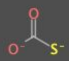	C(=O)([O-])[S-]	p	Hauck, 1980
ZPTA (Thiophosphoryl triamide)	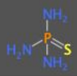	NP(=S)(N)N	p	Radel et al., 1992
Isothiocyanates	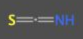	N=C=S	p	Slangen and Kerkhoff, 1984
Potassium azide	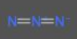	[N-]=[N+]=[N-]	p	Hughes and Welch, 1970
Sodium thiocarbamate	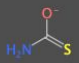	C(=S)(N)[O-]	p	Hauck, 1980
s-ethyl dipropylthiocarbamate	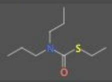	CCCN(CCC)C(=O)SCC	p	Hauck, 1980
Ethylene-bis-dithiocarbamate	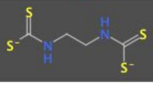	C(CNC(=S)[S-])NC(=S)[S-]	p	Hauck, 1980

Name	Structure of Smiles	Smiles	Activity	Reference
Sodium diethylthiocarbamate	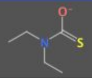	CCN(CC)C(=S)[O-]	p	Bundy and Bremner, 1973
Phenyl mercuric acetate	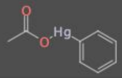	CC(=O)O[Hg]C1=CC=CC=C1	p	Subbarao et al., 2005
2-ethynylpyridine	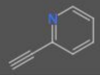	C#CC1=CC=CC=N1	p	McCarty and Bremner, 1986
3-Methylpyrazole-1-carboxamide	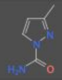	CC1=NN(C=C1)C(=O)N	p	Subbarao et al., 2005
Phenylacetylene	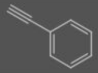	C#CC1=CC=CC=C1	p	McCarty and Bremner, 1986
Propyne	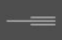	CC#C	p	McCarty and Bremner, 1986
1-Butyne	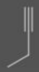	CCC#C	p	McCarty and Bremner, 1986
3-Butyne-2	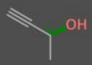	CC(C#C)O	p	McCarty and Bremner, 1986
3-chloro-acetanilide	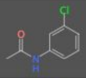	CC(=O)NC1=CC(=CC=C1)Cl	p	Subbarao et al., 2005
2,5-dichloro-aniline	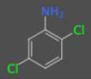	C1=CC(=C(C=C1Cl)N)Cl	p	Bundy and Bremner, 1973
o-nitrophenol	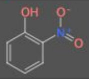	C1=CC=C(C(=C1)[N+](=O)[O-])O	p	Topalova et al., 1995
m-nitroaniline	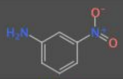	C1=CC(=CC(=C1)[N+](=O)[O-])N	p	Hoeflich, 1968
o-nitroaniline	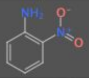	C1=CC=C(C(=C1)N)[N+](=O)[O-]	p	Hermann et al., 1967
Benzotriazole	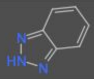	C1=CC2=NNN=C2C=C1	p	McCarty and Bremner, 1989
Pyrazole	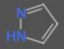	C1=CNN=C1	p	McCarty, 1999
1,2,4-Triazole	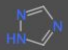	C1=NC=NN1	p	McCarty, 1999
Pyridazine	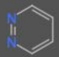	C1=CC=NN=C1	p	McCarty, 1999
Indazole	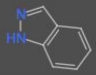	C1=CC=C2C(=C1)C=NN2	p	McCarty, 1999
Carbon monoxide	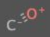	[C-]#[O+]	p	Bremner and Bundy, 1974
Dimethyl ether	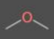	COC	p	Bremner and McCarty, 1993

Name	Structure of Smiles	Smiles	Activity	Reference
Cyclohexane	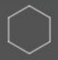	C1CCCCC1	p	Conrad, 1989
Methyl sulfide	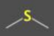	CSC	p	Dacey and Klug, 1979
Ethyl sulfide	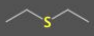	CCSCC	p	Dacey and Klug, 1979
Tetrahydrothiophene	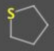	C1CCSC1	p	Dacey and Klug, 1979
Thiophene	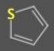	C1=CSC=C1	p	Dacey and Klug, 1979
Methylphenylsulfide	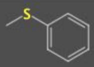	CSC1=CC=CC=C1	p	Dacey and Klug, 1979
Allylmethyl sufide	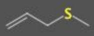	CSCC=C	p	Dacey and Klug, 1979
Allysulfide	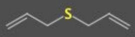	C=CCSCC=C	p	Dacey and Klug, 1979
Bromoethane	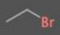	CCBr	p	Dalvi et al., 1974
Fluoroethane	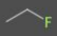	CCF	p	Drozd, 1980
Dichloromethane	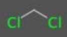	C(Cl)Cl	p	Drozd, 1980
Chloroethane	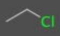	CCCl	p	Dalvi et al., 1974
1,1-Dichloroethane	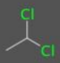	CC(Cl)Cl	p	Drozd, 1980
1,2-Dichloroethane	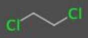	C(CCl)Cl	p	Drozd, 1980
1,1,1-Trichloroethane	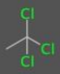	CC(Cl)(Cl)Cl	p	Drozd, 1980
1,1,2-Trichloroethane	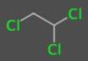	C(C(Cl)Cl)Cl	p	Drozd, 1980
1,1,1,2-Tetrachloroethane	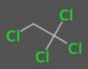	C(C(Cl)(Cl)Cl)Cl	p	Drozd, 1980
Chloropropane	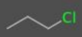	CCCCl	p	Dalvi et al., 1974
1,2-Dichloropropane	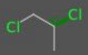	CC(CCl)Cl	p	Dalvi et al., 1974
Chlorobutane	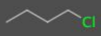	CCCCCl	p	Dalvi et al., 1974

Name	Structure of Smiles	Smiles	Activity	Reference
Chloroethylene	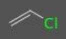	C=CCl	p	Drozd, 1980
Trichloroethylene	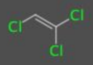	C(=C(Cl)Cl)Cl	p	Dunfield and Knowles, 1995
Benzene	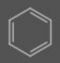	C1=CC=CC=C1	p	Ensign et al., 1993
Toluene	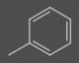	CC1=CC=CC=C1	p	Ensign et al., 1993
Ethylbenzene	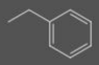	CCC1=CC=CC=C1	p	Ensign et al., 1993
p-Xylene	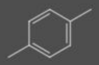	CC1=CC=C(C=C1)C	p	Ensign et al., 1993
Styrene	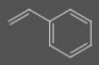	C=CC1=CC=CC=C1	p	Ensign et al., 1993
Bromobenzene	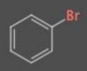	C1=CC=C(C=C1)Br	p	Ensign et al., 1993
Iodobenzene	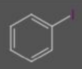	C1=CC=C(C=C1)I	p	Ensign et al., 1993
1,2-Dichlorobenzene	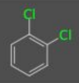	C1=CC=C(C(=C1)Cl)Cl	p	Ensign et al., 1993
Nitrobenzene	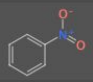	C1=CC=C(C=C1)[N+](=O)[O-]	p	Ensign et al., 1993
Acetophenone	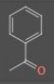	CC(=O)C1=CC=CC=C1	p	Ensign et al., 1993
Benzylalcohol	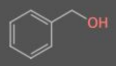	C1=CC=C(C=C1)CO	p	Ensign et al., 1993
p-Methylbenzyl alcohol	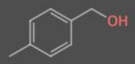	CC1=CC=C(C=C1)CO	p	Ensign et al., 1993
guanylthiourea	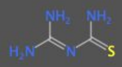	C(=NC(=S)N)(N)N	p	Hauck, 1980
dithiocarbamate	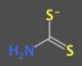	C(=S)(N)[S-]	p	Hauck, 1980
dimethyl disulfide	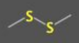	CSSC	p	Bremner and Bundy, 1974
acetylene monocarboxylic acid	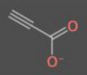	C#CC(=O)[O-]	n	McCarty, 1999
2-butynoic acid	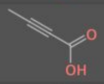	CC#CC(=O)O	n	McCarty, 1999
Benzimidazole	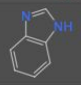	C1=CC=C2C(=C1)NC=N2	n	McCarty, 1999

Name	Structure of Smiles	Smiles	Activity	Reference
Imidazole	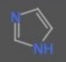	C1=CN=CN1	n	McCarty, 1999
Pyrrole	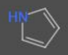	C1=CNC=C1	n	McCarty, 1999
Pyrimidine	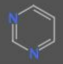	C1=CN=CN=C1	n	McCarty, 1999
s-Triazine	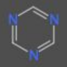	C1=NC=NC=N1	n	McCarty, 1999
Pyridine	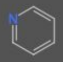	C1=CC=NC=C1	n	McCarty, 1999
Indole	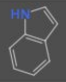	C1=CC=C2C(=C1)C=CN2	n	McCarty, 1999
Alloxydim	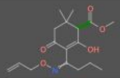	CCCC(=NOCC=C)C1=C(C(C(CC1=O)(C)C)C(=O)OC)O	n	Pell et al., 1998
Bentazone	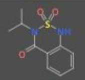	CC(C)N1C(=O)C2=CC=CC=C2NS1(=O)=O	n	Pell et al., 1998
Bromacil	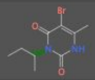	CCC(C)N1C(=O)C(=C(NC1=O)C)B[illegible]	n	Pell et al., 1998
Chlorbromuron	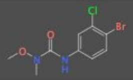	CN(C(=O)NC1=CC(=C(C=C1)Br)Cl)OC	n	Pell et al., 1998
Dalapon-Na	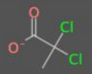	CC(C(=O)[O-])(Cl)Cl	n	Pell et al., 1998
2,4-D	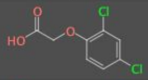	C1=CC(=C(C=C1Cl)Cl)OCC(=O)O	n	Pell et al., 1998
2,4-DB	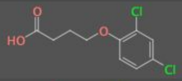	C1=CC(=C(C=C1Cl)Cl)OCCCC(=O)O	p	Pell et al., 1998
2,4-DP	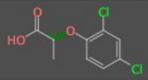	CC(C(=O)O)OC1=C(C=C(C=C1)Cl)Cl	n	Pell et al., 1998
Diuron	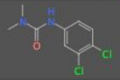	CN(C)C(=O)NC1=CC(=C(C=C1)Cl)Cl	n	Pell et al., 1998
Glyphosate iso-propylamid	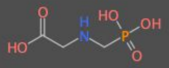	C(C(=O)O)NCP(=O)(O)O	n	Pell et al., 1998
Hexazinone	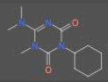	CN1C(=NC(=O)N(C1=O)C2CCCCC2)N(C)C	n	Pell et al., 1998
Imazapyr	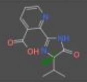	CC(C)C1(C(=O)NC(=N1)C2=C(C=CC=N2)C(=O)O)C	n	Pell et al., 1998
Ioxynil	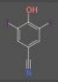	C1=C(C=C(C(=C1I)O)I)C#N	p	Pell et al., 1998
Lenacil	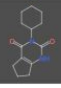	C1CCC(CC1)N2C(=O)C3=C(CCC3)NC2=O	n	Pell et al., 1998

Name	Structure of Smiles	Smiles	Activity	Reference
Linuron	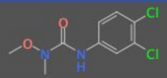	CN(C(=O)NC1=CC(=C(C=C1)Cl)Cl)OC	n	Pell et al., 1998
MCPA	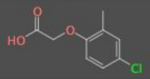	CC1=C(C=CC(=C1)Cl)OCC(=O)O	n	Pell et al., 1998
Benomyl	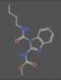	CCCCNC(=O)N1C2=CC=CC=C2N=C 1NC(=O)OC	n	Pell et al., 1998
Carbendazim	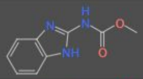	COC(=O)NC1=NC2=CC=CC=C2N1	n	Pell et al., 1998
Iprodione	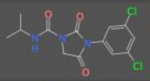	CC(C)NC(=O)N1CC(=O)N(C1=O)C2= CC(=CC(=C2)Cl)Cl	n	Pell et al., 1998
Maneb	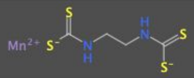	C(CNC(=S)[S-])NC(=S)[S-].[Mn+2]	p	Pell et al., 1998
Aldrin	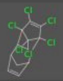	C1C2C=CC1C3C2C4(C(=C(C3(C4(Cl) Cl)Cl)Cl)Cl)Cl	n	Pell et al., 1998
Cyromazine	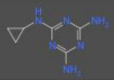	C1CC1NC2=NC(=NC(=N2)N)N	n	Pell et al., 1998
Fenvalerate	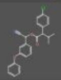	CC(C)C(C1=CC=C(C=C1)Cl)C(=O)OC(C#N)C2=CC(=CC=C2)OC3=CC=CC= C3	n	Pell et al., 1998
MCPB	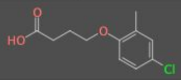	CC1=C(C=CC(=C1)Cl)OCCCC(=O)O	n	Pell et al., 1998
MCPP	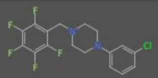	C1CN(CCN1CC2=C(C(=C(C(=C2F)F)F) F)F)C3=CC(=CC=C3)Cl	n	Pell et al., 1998
Metobromuron	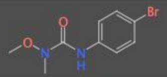	CN(C(=O)NC1=CC=C(C=C1)Br)OC	n	Pell et al., 1998
Metribuzin	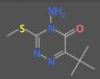	CC(C)(C)C1=NN=C(N(C1=O)N)SC	n	Pell et al., 1998
Napropamide	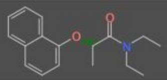	CCN(CC)C(=O)C(C)OC1=CC=CC2=C C=CC=C21	n	Pell et al., 1998
Picloram	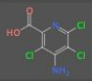	C1(=C(C(=NC(=C1Cl)Cl)C(=O)O)Cl)N	n	Pell et al., 1998
Propyzamide	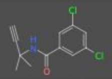	CC(C)(C#C)NC(=O)C1=CC(=CC(=C1) Cl)Cl	n	Pell et al., 1998
Simazine	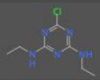	CCNC1=NC(=NC(=N1)Cl)NCC	n	Pell et al., 1998
TBA	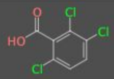	C1=CC(=C(C(=C1Cl)C(=O)O)Cl)C	n	Pell et al., 1998
TCA	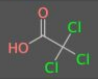	C(=O)(C(Cl)(Cl)Cl)O	n	Pell et al., 1998
2,4,5-T	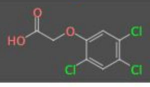	C1=C(C(=CC(=C1Cl)Cl)Cl)OCC(=O)O	n	Pell et al., 1998

Name	Structure of Smiles	Smiles	Activity	Reference
Terbuthylazine	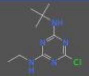	CCNC1=NC(=NC(=N1)Cl)NC(C)(C)C	n	Pell et al., 1998
Terbutryn	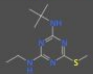	CCNC1=NC(=NC(=N1)SC)NC(C)(C)C	n	Pell et al., 1998
Tri-allat	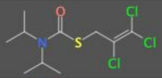	CC(C)N(C(C)C)C(=O)SCC(=C(Cl)Cl)Cl	n	Pell et al., 1998
Triclopyr	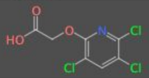	C1=C(C(=NC(=C1Cl)Cl)OCC(=O)O)Cl	n	Pell et al., 1998
Trifluralin	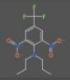	CCCN(CCC)C1=C(C=C(C=C1[N+](=O)[O-])C(F)(F)F)[N+](=O)[O-]	n	Pell et al., 1998
Thiophanate-methyl	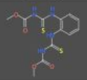	COC(=O)NC(=S)NC1=CC=CC=C1NC(=S)NC(=O)OC	n	Pell et al., 1998
Triadimefon	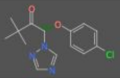	CC(C)(C)C(=O)C(N1C=NC=N1)OC2=CC=C(C=C2)Cl	n	Pell et al., 1998
Triadimenol	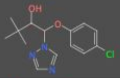	CC(C)(C)C(C(N1C=NC=N1)OC2=CC=C(C=C2)Cl)O	n	Pell et al., 1998
Vinclozolin	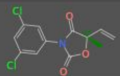	CC1(C(=O)N(C(=O)O1)C2=CC(=CC(=C2)Cl)Cl)C=C	n	Pell et al., 1998
Heptachlor	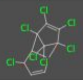	C1=CC(C2C1C3(C(=C(C2(C3(Cl)Cl)Cl)Cl)Cl)Cl)Cl	n	Pell et al., 1998
Permethrin	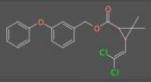	CC1(C(C1C(=O)OCC2=CC(=CC=C2)OC3=CC=CC=C3)C=C(Cl)Cl)C	n	Pell et al., 1998
Linoleic acid	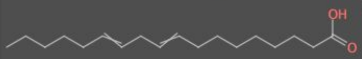	CCCCCC=CCC=CCCCCCCCC(=O)O	p	Subbarao et al., 2008
α-Linolenic acid	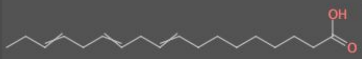	CCC=CCC=CCC=CCCCCCCCC(=O)O	p	Subbarao et al., 2008
Methyl p-coumarate	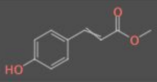	COC(=O)C=CC1=CC=C(C=C1)O	p	Gopalakrishnan et al., 2007
Methyl ferulate	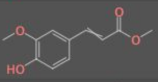	COC1=C(C=CC(=C1)C=CC(=O)OC)O	p	Gopalakrishnan et al., 2007
Methyl 3-(4-hydroxyphenyl) propionate	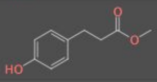	COC(=O)CCC1=CC=C(C=C1)O	p	Zakir et al., 2008
Karanjin	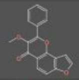	COC1=C(OC2=C(C1=O)C=CC3=C2C=CO3)C4=CC=CC=C4	p	Sahrawat, 1981
trazine-2	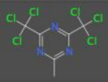	C1(C)N=C(C(Cl)(Cl)Cl)N=C(C(Cl)(Cl)Cl)N=1	p	Takahashi et al., 1997
trazine-3	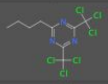	C1(CCCC)N=C(C(Cl)(Cl)Cl)N=C(C(Cl)(Cl)Cl)N=1	p	Takahashi et al., 1997
trazine-6	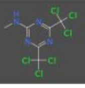	C1(NC)N=C(C(Cl)(Cl)Cl)N=C(C(Cl)(Cl)Cl)N=1	p	Takahashi et al., 1997

Name	Structure of Smiles	Smiles	Activity	Reference
trazine-7	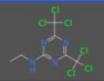	C1(NCC)N=C(C(Cl)(Cl)Cl)N=C(C(Cl)(Cl)Cl)N=1	p	Takahashi et al., 1997
trazine-8	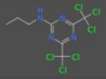	C1(NCCC)N=C(C(Cl)(Cl)Cl)N=C(C(Cl)(Cl)Cl)N=1	p	Takahashi et al., 1997
trazine-9	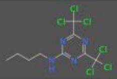	C1(NCCCC)N=C(C(Cl)(Cl)Cl)N=C(C(Cl)(Cl)Cl)N=1	p	Takahashi et al., 1997
trazine-12	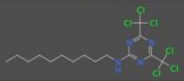	C1(NCCCCCCCCCC)N=C(C(Cl)(Cl)Cl)N=C(C(Cl)(Cl)Cl)N=1	p	Takahashi et al., 1997
trazine-13	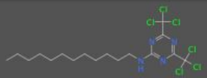	C1(NCCCCCCCCCCCC)N=C(C(Cl)(Cl)Cl)N=C(C(Cl)(Cl)Cl)N=1	p	Takahashi et al., 1997
trazine-14	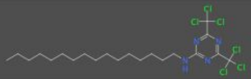	C1(NCCCCCCCCCCCCCCCC)N=C(C(Cl)(Cl)Cl)N=C(C(Cl)(Cl)Cl)N=1	n	Takahashi et al., 1997
trazine-17	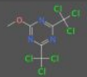	C1(OC)N=C(C(Cl)(Cl)Cl)N=C(C(Cl)(Cl)Cl)N=1	p	Takahashi et al., 1997
trazine-18	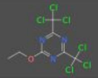	C1(OCC)N=C(C(Cl)(Cl)Cl)N=C(C(Cl)(Cl)Cl)N=1	p	Takahashi et al., 1997
trazine-20	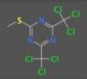	C1(SC)N=C(C(Cl)(Cl)Cl)N=C(C(Cl)(Cl)Cl)N=1	p	Takahashi et al., 1997
trazine-22	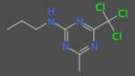	C1(NCCC)N=C(C(Cl)(Cl)Cl)N=C(C)N=1	p	Takahashi et al., 1997
trazine-23	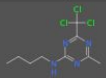	C1(NCCCC)N=C(C(Cl)(Cl)Cl)N=C(C)N=1	p	Takahashi et al., 1997
trazine-24	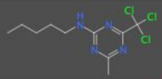	C1(NCCCCC)N=C(C(Cl)(Cl)Cl)N=C(C)N=1	p	Takahashi et al., 1997
trazine-27	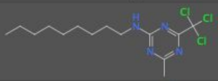	C1(NCCCCCCCCC)N=C(C(Cl)(Cl)Cl)N=C(C)N=1	n	Takahashi et al., 1997
trazine-28	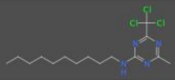	C1(NCCCCCCCCCC)N=C(C(Cl)(Cl)Cl)N=C(C)N=1	n	Takahashi et al., 1997
trazine-29	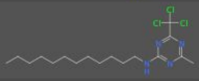	C1(NCCCCCCCCCCCC)N=C(C(Cl)(Cl)Cl)N=C(C)N=1	n	Takahashi et al., 1997
trazine-30	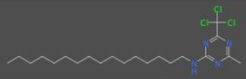	C1(NCCCCCCCCCCCCCCCC)N=C(C(Cl)(Cl)Cl)N=C(C)N=1	n	Takahashi et al., 1997
trazine-32	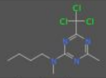	C1(N(C)CCCC)N=C(C(Cl)(Cl)Cl)N=C(C)N=1	p	Takahashi et al., 1997
trazine-33	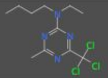	C1(N(CC)CCCC)N=C(C(Cl)(Cl)Cl)N=C(C)N=1	p	Takahashi et al., 1997
trazine-34	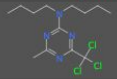	C1(N(CCCC)CCCC)N=C(C(Cl)(Cl)Cl)N=C(C)N=1	p	Takahashi et al., 1997
trazine-38	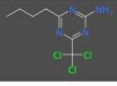	C1(N)N=C(C(Cl)(Cl)Cl)N=C(CCCC)N=1	p	Takahashi et al., 1997

Name	Structure of Smiles	Smiles	Activity	Reference
trazine-39	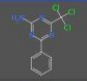	C1(N)N=C(C(Cl)(Cl)Cl)N=C(C2=CC=CC=C2)N=1	p	Takahashi et al., 1997
trazine-40	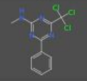	C1(NC)N=C(C(Cl)(Cl)Cl)N=C(C2=CC=CC=C2)N=1	p	Takahashi et al., 1997
trazine-41	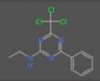	C1(NCC)N=C(C(Cl)(Cl)Cl)N=C(C2=CC=CC=C2)N=1	p	Takahashi et al., 1997
trazine-42	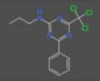	C1(NCCC)N=C(C(Cl)(Cl)Cl)N=C(C2=CC=CC=C2)N=1	p	Takahashi et al., 1997
trazine-44	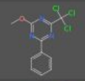	C1(OC)N=C(C(Cl)(Cl)Cl)N=C(C2=CC=CC=C2)N=1	p	Takahashi et al., 1997
trazine-46	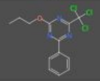	C1(OCCC)N=C(C(Cl)(Cl)Cl)N=C(C2=CC=CC=C2)N=1	p	Takahashi et al., 1997
trazine-47	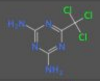	C1(N)N=C(C(Cl)(Cl)Cl)N=C(N)N=1	p	Takahashi et al., 1997
trazine-48	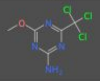	C1(OC)N=C(C(Cl)(Cl)Cl)N=C(N)N=1	p	Takahashi et al., 1997
trazine-49	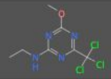	C1(OC)N=C(C(Cl)(Cl)Cl)N=C(NCC)N=1	p	Takahashi et al., 1997
trazine-51	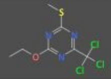	C1(OCC)N=C(C(Cl)(Cl)Cl)N=C(SC)N=1	p	Takahashi et al., 1997
trazine-52	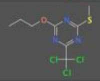	C1(OCCC)N=C(C(Cl)(Cl)Cl)N=C(SC)N=1	p	Takahashi et al., 1997
prosulfuron	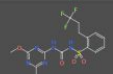	CC1=NC(=NC(=N1)OC)NC(=O)NS(=O)(=O)C2=CC=CC=C2CCC(F)(F)F	p	Arora et al., 2003
chlorothalonil	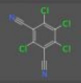	C(#N)C1=C(C(=C(C(=C1Cl)Cl)Cl)C#N)Cl	p	Lang and Cai, 2009
2,6-dihydro-2,2,4-trimethyl-6-quinone imine (QI)	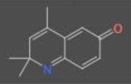	CC1=CC(N=C2C1=CC(=O)C=C2)(C)C	p	Papadopoulou et al., 2020
2,4-dimethyl-6-ethoxy-quinoline (EQNL)	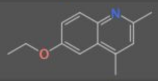	CCOC1=CC2=C(C=C1)N=C(C=C2C)C	p	Papadopoulou et al., 2020
Ethoxyquin (EQ)	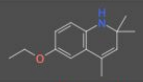	CCOC1=CC2=C(C=C1)NC(C=C2C)(C)C	p	Papadopoulou et al., 2020
Chlorpyrifos	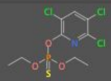	CCOP(=S)(OCC)OC1=NC(=C(C=C1Cl)Cl)Cl	p	Lu et at., 2020
AMPA	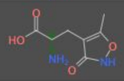	CC1=C(C(=O)NO1)CC(C(=O)O)N	n	Pell et al., 1998
Metsulfuron-methyl	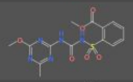	CC1=NC(=NC(=N1)OC)NC(=O)NS(=O)(=O)C2=CC=CC=C2C(=O)OC	p	El-Ghamry, A. M., et al., 2002
Pyraclostrobin	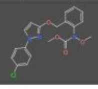	COC(=O)N(C1=CC=CC=C1COC2=NN(C=C2)C3=CC=C(C=C3)Cl)OC	p	Subbarao et al., 2006

Name	Structure of Smiles	Smiles	Activity	Reference
3,5 DCA	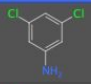	C1=C(C=C(C=C1Cl)Cl)N	p	Vasileiadis et al., 2018
salicylaldoxime	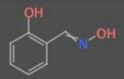	C1=CC=C(C(=C1)C=NO)O	p	Hooper and Terry, 1973
allylsulfide	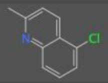	CC1=NC2=C(C=C1)C(=CC=C2)Cl	p	Hyman et al., 1988
Nitrapyrin	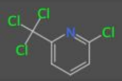	C1=CC(=NC(=C1)Cl)C(Cl)(Cl)Cl	p	Powell and Prosser, 1986
O,O-Diethyl thiophosphate	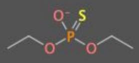	CCOP(=S)([O-])OCC	n	
2-Methyl-4-amino-6-methoxy-s-triazine	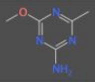	CC1=NC(=NC(=N1)OC)N	n	
Saccharin	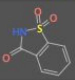	C1=CC=C2C(=C1)C(=O)NS2(=O)=O	n	
Benzoic acid, 2-(aminosulfonyl)- methyl ester	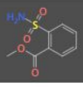	COC(=O)C1=CC=CC=C1S(=O)(=O)N	n	

Compounds reported as commercial nitrification inhibitors and compounds that inhibited nitrification by more than 50% at concentrations of less than 80 mg/L are labeled as positive in the dataset, while the remaining compounds were labeled as negative. The last four compounds are transformation products of pesticides, and they were tested in our own lab with AOB (i.e., *Nitrosospira multiformis*).

2.3 External *In Vitro* Experimental Data

Compound	Structure of Smiles	Smiles	Activity
Quinic acid	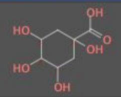	C1C(C(C(CC1(C(=O)O)O)O)O)O	n
MHPP	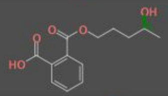	CC(O)CCCOC(=O)C1=CC=CC=C1C(O)=O	n
Shikimic acid	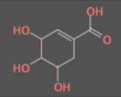	C1C(C(C(C=C1C(=O)O)O)O)O	n
Chlorogenic acid	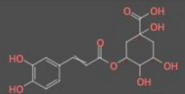	C1C(C(C(CC1(C(=O)O)O)OC(=O)C=CC2=CC(=C(C=C2)O)O)O)O	n
Zeanone	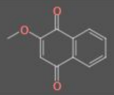	C1=CC2=C(C=C1)C(=O)C=C(C2=O)OC	n
Caffeic acid	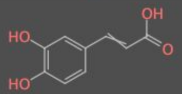	C1=CC(=C(C=C1C=CC(=O)O)O)O	n
2-methoxy-1,4-naphthoc	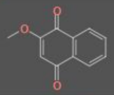	COC1=CC(=O)C2=CC=CC=C2C1=O	n
Sakuranetin	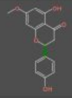	COC1=CC(=C2C(=O)CC(OC2=C1)C3=CC=C(C=C3)O)O	n
Simvastatin	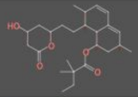	CCC(C(=O)OC1CC(C)C=C2C1C(CCC1CC(O)CC(=O)O1)C(C=C2)C)(C)C	n
Albendazole	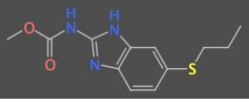	CCCSC1=CC2=C(C=C1)N=C(N2)NC(=O)OC	p

3. Graph Attention Convolution and Multi-Head Global Attention

Graph Attention Convolution

We leveraged GATConv layers to efficiently process molecular data structured in the form of graphs. GATConv layers introduced a localized attention mechanism to dynamically weight the influence of neighboring atoms, allowing for an adaptive feature aggregation process. For a given atom i, the feature representation was updated through a weighted sum of the features (or embeddings) of its neighbors (e.g., atom j), where the weights were determined by an attention mechanism a. The attention coefficients s_{ij} for atom i and atom j were computed as follows:

$$s_{ij} = a(\mathrm{W}\vec{h}_i, \mathrm{W}\vec{h}_j, \mathrm{U}\vec{e}_{ij}) \quad (1)$$

where W and U were weight matrices applied to the atom feature $\vec{h}$ and bond feature $\vec{e}$, respectively. The learnable function a was the attention mechanism that computed the relevance of atom j to atom i with edge attributes $\vec{e}_{ij}$. Normalization of the attention coefficients was achieved through a softmax function applied over all neighbors j of atom i, ensuring that the coefficients sum to one across the neighborhood (eq. 2).

$$\alpha_{ij} = softmax_j(s_{ij}) = \frac{exp(s_{ij})}{\sum_{k\in\mathcal{N}_i} exp(s_{ik})} \quad (2)$$

The updated representation of atom i, $\vec{h'}_i$, was calculated by aggregating the contributions of its neighbors (i.e., $\mathcal{N}_i$) and processed by the activation function $tanh$ (eq. 3).

$$\vec{h'}_i = tanh(\alpha_{ii}\mathrm{W}\vec{h}_i + \sum_{j\in\mathcal{N}_i} \alpha_{ij}\mathrm{W}\vec{h}_j \quad (3)$$

In our GATConv layers, we applied P (P=4) attention heads, which involved P independent attention mechanisms executing in parallel to compute P sets of updated representation. The P updated representations were then concatenated (i.e., $\Vert_{p=1}^{P}$) and linearly transformed by weight matrix M, followed by a batch normalization BN, to produce the final atom representations (eq. 4).

$$\vec{h'}_i = \mathrm{BN}(\mathrm{M} \Vert_{p=1}^{P} \vec{h'}_i^{p} \quad (4)$$

Multi-Head Global Attention

In addition to the localized attention mechanism introduced by GATConv layers, we implemented a multi-head attention mechanism to enable the model to consider the entire molecule. It allowed the model to capture global relationships and interactions within the molecule. The feature Matrix H of atoms from GATConv layers were transformed through separate linear transformations to create queries Q, keys K and values V for each attention head. The embedding size was evenly divided among N attention heads, with each head operating on a fraction of the embedding dimension, leading to an attention size per head denoted as $d_k = Atom\ feature\ size/\mathrm{N}$. The

transformation for queries, keys, and values was defined as follows, where W^Q, W^K, and W^V represented the weight matrices for queries, keys, and values, respectively.

$$\mathrm{Q} = W^Q H, \qquad K = W^K H, \qquad V = W^V \quad (5)$$

These three matrices were reshaped and transposed to align for the multi-head computation, enabling parallel processing across the N heads. Attention weights A were computed using the scaled dot-product attention mechanisms (eq. 6), where the scaling factor normalized the dot product to prevent them from growing too large. The softmax operation ensured that the weights sum to one.

$$\mathrm{A} = \mathrm{softmax}\left(\frac{QK^T}{\sqrt{d_k}}\right) \quad (6)$$

The output of the attention mechanism for each head was then calculated as the weighted sum of the values, scaled by the attention weights (eq. 7).

$$head_i = \quad (7)$$

Subsequently, outputs from all heads were concatenated (i.e., $\Vert_{i=1}^{\mathrm{N}}$) and linearly transformed by W^O to produce the final output, followed by a layer normalization LN.

$$\mathrm{MultiHead(Q,K,V)} = \mathrm{LN}(W^O \Vert_{i=1}^{\mathrm{N}} head_i \quad (8)$$

The output of the multi-head attention was added with the feature matrix H of atoms, yielding an updated matrix H′. This resultant matrix H′was subsequently used as input to two fully connected layers for the prediction of molecular properties.

$$\mathrm{H}' = \mathrm{H} + \mathrm{MultiHead(Q,K,V)} \quad (9)$$

4. KOWWIN Coefficients and Shapley Values

This section contains the comparison of Shapley values and KOWWIN coefficients for seven compounds with logP values ranging from -4.22 to 5.42. Substructures contributing significantly to logP values are highlighted based on the absolute values of their KOWWIN coefficients.

To systematically quantify the similarity or correlation between Shapley values and KOWWIN coefficients, we selected 100 compounds whose logP values predicted by the KOWWIN tool were within 20% of their experimental logP values. This criterion ensured the reliability of KOWWIN predictions and the structural coefficients proposed by KOWWIN. For each of 100 compounds, we recorded the coefficients for each structure provided by KOWWIN and calculated the corresponding Shapley values. We applied kernel density estimation (KDE) to visualize the smoothed distributions of normalized Shapley values and KOWWIN coefficients. Additionally, we calculated the area under the overlap of the KDEs as a robust quantitative measure of similarity. An overlap area of 0 indicates that the two distributions are completely disjoint, whereas an overlap area of 1 indicates perfect overlap, suggesting that the two distributions are identical.

Smiles	O=C(O)C(N)CCCN
logP (exp)	-4.22
logP (KOWWIN)	-3.48
logP (GAT)	-4.1

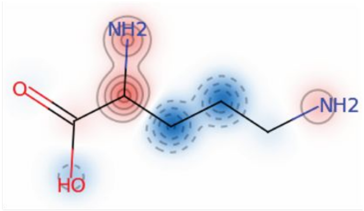

Fragment/Factor	Number	Coefficient	Shapley Value
-CH2- [aliphatic carbon]	3	0.49	>0
-CH [aliphatic carbon]	1	0.36	>0
-NH2 [aliphatic attach]	2	-1.41	<0
-COOH [acid, aliphatic attach]	1	-0.69	<0
Amino acid (alpha-position) correction	1	-2.02	<0
Equation Constant	0	0	/

Figure S10. Comparison of KOWWIN coefficients and Shapley values for the compound with a logP -4.22.

Smiles	CC(=O)NCC(=O)NC (C)C(=O)NC(C(C)C)C(=O)N
logP (exp)	-1.56
logP (KOWWIN)	-1.90
logP (GAT)	-1.49

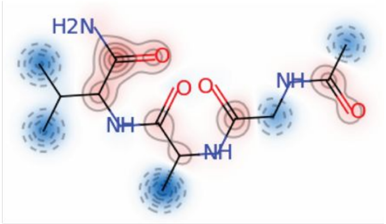

Fragment/Factor	Number	Coefficient	Shapley Value
-CH3 [aliphatic carbon]	4	0.55	>0
-CH2- [aliphatic carbon]	1	0.49	>0
-CH [aliphatic carbon]	3	0.36	>0
-NH2 [aliphatic attach]	1	-1.41	<0
-NH- [aliphatic attach]	3	-1.50	<0
-C(=O)N [aliphatic attach]	4	-0.52	<0
-CO-N-C-CO-N- (linear C;aliphatic) corr.	3	0.7	>0
Equation Constant	0	0	/

Figure S11. Comparison of KOWWIN coefficients and Shapley values for the compound with a logP -1.56.

Smiles	CS(=O)C
logP (exp)	-1.35
logP (KOWWIN)	-1.22
logP (GAT)	-1.00

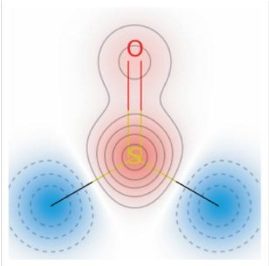

Fragment/Factor	Number	Coefficient	Shapley Value
-CH3 [aliphatic carbon]	2	0.55	>0
-S(=O)- [sulfoxide, aliphatic attach]	1	-2.54	<0
Equation Constant	0	0	/

Figure S12. Comparison of KOWWIN coefficients and Shapley values for the compound with a logP -1.35.

Smiles	ONC(=O)c1ccccc1
logP (exp)	0.26
logP (KOWWIN)	0.61
logP (GAT)	0.35

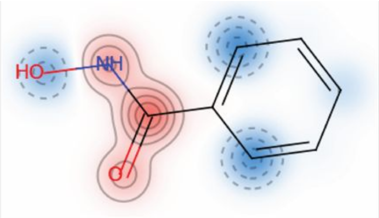

Fragment/Factor	Number	Coefficient	Shapley Value
--NH- [aliphatic attach]	1	-1.50	<0
Aromatic Carbon	6	0.29	>0
-C(=O)N [aromatic attach]	1	0.16	>0
-OH [hydroxy, nitrogen attach]	1	-0.04	<0
Equation Constant	0	0	/

Figure S13. Comparison of KOWWIN coefficients and Shapley values for the compound with a logP 0.26.

Smiles	Cc1ccc(N)nc1
logP (exp)	1.02
logP (KOWWIN)	1.08
logP (GAT)	0.91

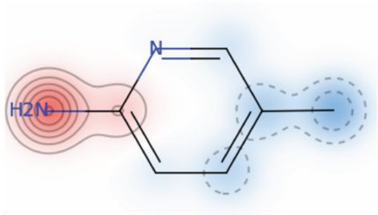

Fragment/Factor	Number	Coefficient	Shapley Value
-CH2- [aliphatic carbon]	1	0.55	>0
Aromatic Carbon	5	0.29	>0
Aromatic Nitrogen	1	-0.73	<0
-N [aliphatic N, one aromatic attach]	1	-0.92	<0
Ortho-Amino pyridine correction	1	0.64	>0
Pyridine ring (non-fused) correction	1	-0.16	<0
Equation COnstant	0	0	/

Figure S14. Comparison of KOWWIN coefficients and Shapley values for the compound with a logP 1.02.

Smiles	c1cc(SC)ccc1NS(=O)(=O)C(F)(F)F
logP (exp)	3.74
logP (KOWWIN)	3.68
logP (GAT)	3.63

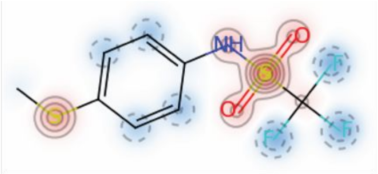

Fragment/Factor	Number	Coefficient	Shapley Value
-CH3 [aliphatic carbon]	1	0.55	>0
C [aliphatic carbon - No H, not tert]	1	0.97	>0
-F [fluorine, aliphatic attach]	3	-0.003	<0
-Aromatic Carbon	6	0.29	>0
-N [aliphatic N, one aromatic attach]	1	-0.92	<0
-S- [aliphatic sulfur,one aromatic attach]	1	0.05	>0
-SO2-N [aliphatic attach]	1	-0.43	<0
SO-C(polyhalo) structure correction	1	1.48	<0
Equation Constant	0	0	/

Figure S15. Comparison of KOWWIN coefficients and Shapley values for the compound with a logP 3.74.

Smiles	Clc1ccc(cc1)c1cccc(Cl)c1Cl
logP (exp)	5.42
logP (KOWWIN)	5.69
logP (GAT)	5.53

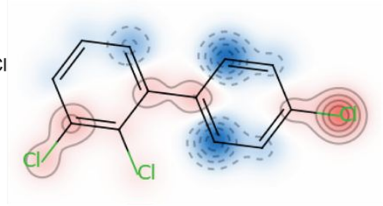

Fragment/Factor	Number	Coefficient	Shapley Value
Aromatic Carbon	12	0.29	>0
-Cl [chlorine, aromatic attach]	3	0.64	>0
Equation Constant	0	0	/

Figure S16. Comparison of KOWWIN coefficients and Shapley values for the compound with a logP 5.42.

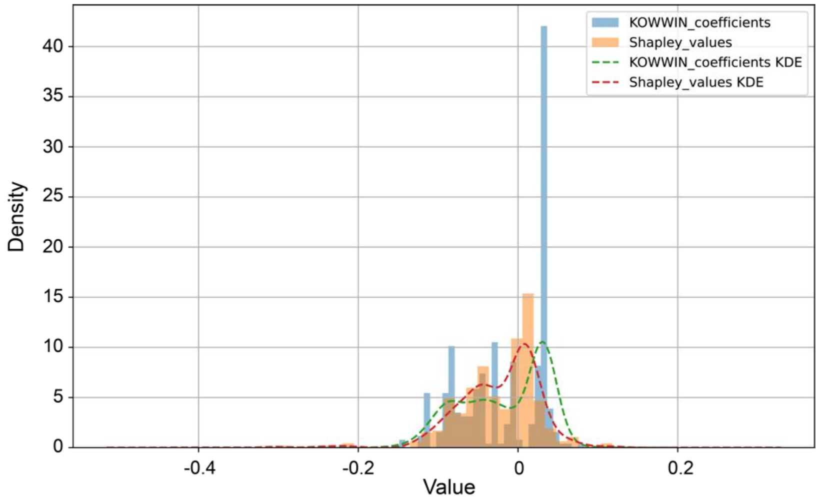

Figure S17. Distribution of KOWWIN coefficients and calculated Shapley values with KDE overlays. The area under the overlap of KDEs is 0.8.

5. Structural Alerts

Table S6: Nitrogen containing structural alerts.

Structural Alerts	Contribution to logP prediction	Contribution to tox prediction
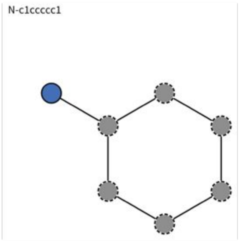	Negative	Positive
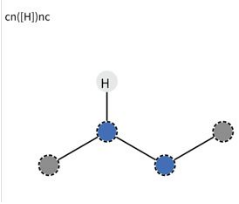	Negative	Positive
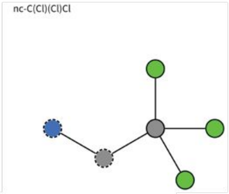	Negative	Positive
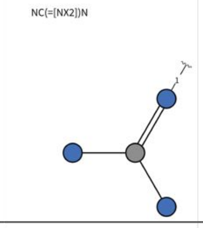	Negative	Positive
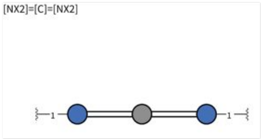	Negative	Positive
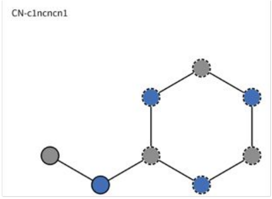	Negative	Positive
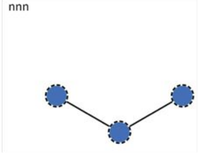	Negative	Positive

Table S7: Sulfur containing structural alerts.

Structural Alerts	Contribution to logP prediction	Contribution to tox prediction
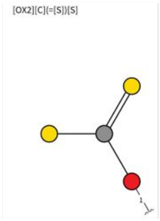	Positive	Positive
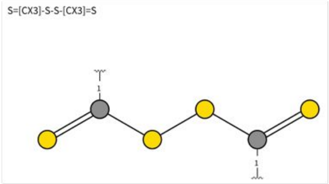	Positive	Positive
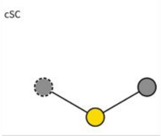	Positive	Positive
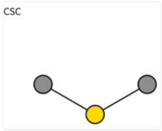	Positive	Positive
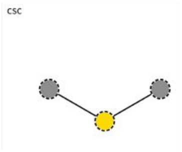	Positive	Positive

Table S8. Nitrogen-sulfur containing structural alerts.

Structural Alerts	Contribution to logP prediction	Contribution to tox prediction
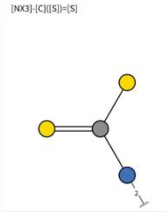	Positive	Positive
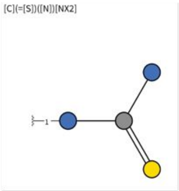	Negative	Positive
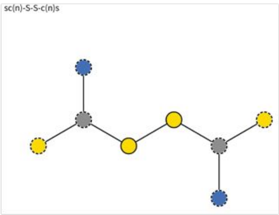	Positive	Positive
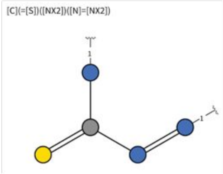	Positive	Positive
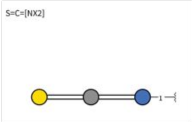	Positive	Positive
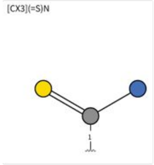	Positive	Positive
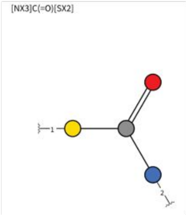	Positive	Positive

Table S9. Halogen containing structural alerts.

Structural Alerts	Contribution to logP prediction	Contribution to tox prediction
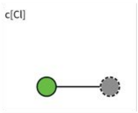	Positive	Positive
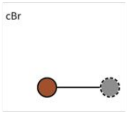	Positive	Positive
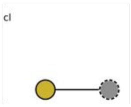	Positive	Positive

Table S10. Other structural alerts.

Structural Alerts	Contribution to logP prediction	Contribution to tox prediction
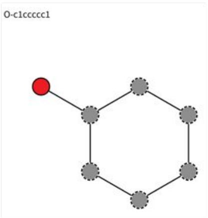	Negative	Positive
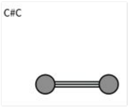	Positive	Positive

References

1. Ulrich, N.; Goss, K. U.; Ebert, A., Exploring the octanol-water partition coefficient dataset using deep learning techniques and data augmentation. *Commun Chem* **2021,** *4* (1), 90.
2. Ramsundar, B.; Pande, V.; Eastman, P.; Feinberg, E.; Gomes, J.; Leswing, K.; Pappu, A.; Wu, M., Democratizing deep-learning for drug discovery, quantum chemistry, materials science and biology. *GitHub repository* **2016**.
3. Hsu, K.-H.; Su, B.-H.; Tu, Y.-S.; Lin, O. A.; Tseng, Y. J., Mutagenicity in a molecule: identification of core structural features of mutagenicity using a scaffold analysis. *PloS one* **2016,** *11* (2), e0148900.
4. Gaulton, A.; Bellis, L. J.; Bento, A. P.; Chambers, J.; Davies, M.; Hersey, A.; Light, Y.; McGlinchey, S.; Michalovich, D.; Al-Lazikani, B., ChEMBL: a large-scale bioactivity database for drug discovery. *Nucleic acids research* **2012,** *40* (D1), D1100-D1107.
5. Wu, Z.; Ramsundar, B.; Feinberg, E. N.; Gomes, J.; Geniesse, C.; Pappu, A. S.; Leswing, K.; Pande, V., MoleculeNet: a benchmark for molecular machine learning. *Chemical science* **2018,** *9* (2), 513-530.
6. Bremner, J.; Bundy, L., Inhibition of nitrification in soils by volatile sulfur compounds. *Soil Biology and Biochemistry* **1974,** *6* (3), 161-165.
7. Bremner, J. M.; McCarty, G. W., Inhibition of nitrification in soil by allelochemicals derived from plants and plant residues. In *Soil biochemistry*, CRC Press: 2021; pp 181-218.
8. Conrad, R., Control of methane production in terrestrial ecosystems. **1989**.
9. Dacey, J.; Klug, M., Methane efflux from lake sediments through water lilies. *Science* **1979,** *203* (4386), 1253-1255.
10. Dalvi, R. R.; Poore, R. E.; Neal, R. A., Studies of the metabolism of carbon disulfide by rat liver microsomes. *Life Sciences* **1974,** *14* (9), 1785-1796.
11. Drozd, J., Respiration in the ammonia-oxidizing chemoautotrophic bacteria. *Diversity of bacterial respiratory systems* **2018**, 87-112.
12. Dunfield, P.; Knowles, R., Kinetics of inhibition of methane oxidation by nitrate, nitrite, and ammonium in a humisol. *Applied and Environmental Microbiology* **1995,** *61* (8), 3129-3135.
13. Ensign, S. A.; Hyman, M. R.; Arp, D. J., In vitro activation of ammonia monooxygenase from Nitrosomonas europaea by copper. *Journal of bacteriology* **1993,** *175* (7), 1971-1980.
14. Pell, M.; Stenberg, B.; Torstensson, L., Potential denitrification and nitrification tests for evaluation of pesticide effects in soil. *Ambio* **1998**, 24-28.
15. Subbarao, G. V.; Nakahara, K.; Ishikawa, T.; Yoshihashi, T.; Ito, O.; Ono, H.; Ohnishi-Kameyama, M.; Yoshida, M.; Kawano, N.; Berry, W., Free fatty acids from the pasture grass Brachiaria humidicola and one of their methyl esters as inhibitors of nitrification. *Plant and Soil* **2008,** *313*, 89-99.
16. Gopalakrishnan, S.; Subbarao, G. V.; Nakahara, K.; Yoshihashi, T.; Ito, O.; Maeda, I.; Ono, H.; Yoshida, M., Nitrification inhibitors from the root tissues of Brachiaria humidicola, a tropical grass. *Journal of agricultural and food chemistry* **2007,** *55* (4), 1385-1388.
17. Zakir, H. A.; Subbarao, G. V.; Pearse, S. J.; Gopalakrishnan, S.; Ito, O.; Ishikawa, T.; Kawano, N.; Nakahara, K.; Yoshihashi, T.; Ono, H., Detection, isolation and characterization of a root-exuded compound, methyl 3-(4-hydroxyphenyl) propionate, responsible for biological nitrification inhibition by sorghum (Sorghum bicolor). *New Phytologist* **2008,** *180* (2), 442-451.
18. Sahrawat, K., Comparison of karanjin with other nitrification inhibitors for retardation of nitrification of urea N in soil. *Plant and Soil* **1981,** *59*, 495-498.

19. Takahashi, I.; Ohki, S.; Murakami, M.; Takagi, S.; Sato, Y.; Vonk, J. W.; Wakabayashi, K., Mode of action and QSAR studies of nitrification inhibitors: Effect of trichloromethyl-1, 3, 5-triazines on ammonia-oxidizing bacteria. *JOURNAL OF PESTICIDE SCIENCE-PESTICIDE SCIENCE SOCIETY OF JAPAN-JAPANESE EDITION-* **1997,** *22*, 27-32.
20. Arora, S.; Sahni, D., Pesticides effect on soil microbial ecology and enzyme activity-An overview. *Journal of Applied and Natural Science* **2016,** *8* (2), 1126-1132.
21. Man, L.; Zucong, C., Effects of chlorothalonil and carbendazim on nitrification and denitrification in soils. *Journal of Environmental Sciences* **2009,** *21* (4), 458-467.
22. Papadopoulou, E. S.; Bachtsevani, E.; Lampronikou, E.; Adamou, E.; Katsaouni, A.; Vasileiadis, S.; Thion, C.; Menkissoglu-Spiroudi, U.; Nicol, G. W.; Karpouzas, D. G., Comparison of novel and established nitrification inhibitors relevant to agriculture on soil ammonia-and nitrite-oxidizing isolates. *Frontiers in Microbiology* **2020,** *11*, 581283.
23. Lu, C.; Yang, Z.; Liu, J.; Liao, Q.; Ling, W.; Waigi, M. G.; Odinga, E. S., Chlorpyrifos inhibits nitrogen fixation in rice-vegetated soil containing Pseudomonas stutzeri A1501. *Chemosphere* **2020,** *256*, 127098.
24. El-Ghamry, A.; Xu, J.; Huang, C.; Gan, J., Microbial response to bensulfuron-methyl treatment in soil. *Journal of Agricultural and Food Chemistry* **2002,** *50* (1), 136-139.
25. Vasileiadis, S.; Puglisi, E.; Papadopoulou, E.; Pertile, G.; Suciu, N.; Pappolla, R.; Tourna, M.; Karas, P.; Papadimitriou, F.; Kasiotakis, A., Blame it on the metabolite: 3, 5-dichloroaniline rather than the parent compound is responsible for the decreasing diversity and function of soil microorganisms. *Applied and Environmental Microbiology* **2018,** *84* (22), e01536-18.
26. Subbarao, G.; Ito, O.; Sahrawat, K.; Berry, W.; Nakahara, K.; Ishikawa, T.; Watanabe, T.; Suenaga, K.; Rondon, M.; Rao, I. M., Scope and strategies for regulation of nitrification in agricultural systems—challenges and opportunities. *Critical reviews in plant sciences* **2006,** *25* (4), 303-335.
27. Hooper, A. B.; Terry, K. R., Specific inhibitors of ammonia oxidation in Nitrosomonas. *Journal of Bacteriology* **1973,** *115* (2), 480-485.
28. Hyman, M. R.; Murton, I. B.; Arp, D. J., Interaction of ammonia monooxygenase from Nitrosomonas europaea with alkanes, alkenes, and alkynes. *Applied and environmental microbiology* **1988,** *54* (12), 3187-3190.
29. Powell, S. J.; Prosser, J. I., Effect of copper on inhibition by nitrapyrin of growth of Nitrosomonas europaea. *Current Microbiology* **1986,** *14*, 177-179.
30. Rajendra Prasad, R. P.; Power, J., Nitrification inhibitors for agriculture, health, and the environment. **1995**.
31. Ranney, M., Nitrification and urease inhibitors. *Fertilizer additives and soil conditions* **1978**, 168-169.
32. Guthrie, T.; Bomke, A., Nitrification inhibition by N-Serve and ATC in soils of varying texture. *Soil Science Society of America Journal* **1980,** *44* (2), 314-320.
33. GV, S., Root exudates of Brachiaria humidicola inhibit nitrification characterization and quantification of this unique biological phenomenon. *Plant nutrition for food security, human health and environmental protection* **2005**, 444-445.
34. Chemicals, M. T., Mitsui Toatsu ST-nitrification inhibitor. *Techn. Bull* **1968,** *1* (7).
35. Mosier, A.; Duxbury, J.; Freney, J.; Heinemeyer, O.; Minami, K. In *Nitrous oxide emissions from agricultural fields: Assessment, measurement and mitigation*, Progress in

Nitrogen Cycling Studies: Proceedings of the 8th Nitrogen Workshop held at the University of Ghent, 5–8 September, 1994, Springer: 1996; pp 589-602.
36. McCarty, G.; Bremner, J., Inhibition of nitrification in soil by heterocyclic nitrogen compounds. *Biology and Fertility of Soils* **1989,** *8*, 204-211.
37. Goos, R., Identification of ammonium thiosulfate as a nitrification and urease inhibitor. *Soil Science Society of America Journal* **1985,** *49* (1), 232-235.
38. Varsa, E.; SL, L.; Kapusta, G., THE EFFECT OF NITRIFICATION INHIBITORS ON WHEAT YIELD AND SOIL NITROGEN RETENTION. **1981**.
39. Hauck, R. D., Mode of action of nitrification inhibitors. *Nitrification inhibitors—potentials and limitations* **1980,** *38*, 19-32.
40. Radel, R.; Randle, A.; Gautney, J.; Bock, B.; Williams, H., Thiophosphoryl triamide: A dual purpose urease/nitrification inhibitor. *Fertilizer research* **1992,** *31*, 275-280.
41. Slangen, J.; Kerkhoff, P., Nitrification inhibitors in agriculture and horticulture: a literature review. *Fertilizer research* **1984,** *5*, 1-76.
42. Hughes, T.; Welch, L., 2-Chloro-6-(Trichloromethyl) Pyridine as a Nitrification Inhibitor for Anhydrous Ammonia Applied in Different Seasons 1. *Agronomy journal* **1970,** *62* (6), 821-824.
43. Bundy, L.; Bremner, J., Inhibition of nitrification in soils. *Soil Science Society of America Journal* **1973,** *37* (3), 396-398.
44. McCarty, G.; Bremner, J., Inhibition of nitrification in soil by acetylenic compounds. *Soil Science Society of America Journal* **1986,** *50* (5), 1198-1201.
45. Topalova, Y.; Ribarova, I.; Dimkov, R.; Arsov, R., Microbiological features of nitrification-denitrification biological excess phosphorus removal systems in the presence of oNP as an inhibitor. *Water Science and Technology* **1995,** *32* (7), 103-110.
46. Hoeflich, G., Effect of several chemical substances on nitrification, some soil microorganisms, and N leaching. *Albrecht-Thaer-Arcli* **1968,** *12*, 691-699.
47. Hermann, A.; Susanne, M.; Rudolf, J., Inhibition of nitrification by some chemical preparations. *Albrecht-Thaer-Arch* **1967,** *11*, 509-516.
48. McCarty, G., Modes of action of nitrification inhibitors. *Biology and Fertility of Soils* **1999,** *29*, 1-9.
49. Clark, C.; Schmidt, E., Uptake and utilization of amino acids by resting cells of Nitrosomonas europaea. *Journal of Bacteriology* **1967,** *93* (4), 1309-1315.
50. Hockenbury, M. R.; Grady Jr, C. L.; Daigger, G. T., Factors affecting nitrification. *Journal of the Environmental Engineering Division* **1977,** *103* (1), 9-19.
51. Lees, H.; Quastel, J., Biochemistry of nitrification in soil: 3. Nitrification of various organic nitrogen compounds. *Biochemical Journal* **1946,** *40* (5-6), 824.
52. Lees, H.; Simpson, J., The biochemistry of the nitrifying organisms. 5. Nitrite oxidation by Nitrobacter. *Biochemical Journal* **1957,** *65* (2), 297.
53. Tomlinson, T.; Boon, A.; Trotman, C., Inhibition of nitrification in the activated sludge process of sewage disposal. *Journal of Applied Microbiology* **1966,** *29* (2), 266-291.
54. ZAVARZIN, G., On the Inducer of the Second Phase of Nitrification. *Mikrobiologiya* **1958,** *27*, 395-399.
